# Parvovirus Induced Alterations in Nuclear Architecture and Dynamics

**DOI:** 10.1371/journal.pone.0005948

**Published:** 2009-06-17

**Authors:** Teemu O. Ihalainen, Einari A. Niskanen, Juulia Jylhävä, Outi Paloheimo, Nicolas Dross, Hanna Smolander, Jörg Langowski, Jussi Timonen, Maija Vihinen-Ranta

**Affiliations:** 1 NanoScience Center, Department of Biological and Environmental Science, University of Jyväskylä, Jyväskylä, Finland; 2 Department of Microbiology and Immunology, Medical School, University of Tampere, Tampere, Finland; 3 Division Biophysics of Macromolecules, German Cancer Research Center (DKFZ), Heidelberg, Germany; 4 Department of Virology, Haartman Institute, University of Helsinki, Helsinki, Finland; 5 Department of Physics, University of Jyväskylä, Jyväskylä, Finland; Brunel University, United Kingdom

## Abstract

The nucleus of interphase eukaryotic cell is a highly compartmentalized structure containing the three-dimensional network of chromatin and numerous proteinaceous subcompartments. DNA viruses induce profound changes in the intranuclear structures of their host cells. We are applying a combination of confocal imaging including photobleaching microscopy and computational methods to analyze the modifications of nuclear architecture and dynamics in parvovirus infected cells. Upon canine parvovirus infection, expansion of the viral replication compartment is accompanied by chromatin marginalization to the vicinity of the nuclear membrane. Dextran microinjection and fluorescence recovery after photobleaching (FRAP) studies revealed the homogeneity of this compartment. Markedly, in spite of increase in viral DNA content of the nucleus, a significant increase in the protein mobility was observed in infected compared to non-infected cells. Moreover, analyzis of the dynamics of photoactivable capsid protein demonstrated rapid intranuclear dynamics of viral capsids. Finally, quantitative FRAP and cellular modelling were used to determine the duration of viral genome replication. Altogether, our findings indicate that parvoviruses modify the nuclear structure and dynamics extensively. Intranuclear crowding of viral components leads to enlargement of the interchromosomal domain and to chromatin marginalization via depletion attraction. In conclusion, parvoviruses provide a useful model system for understanding the mechanisms of virus-induced intranuclear modifications.

## Introduction

The nuclear replication strategies of DNA viruses and the virus-induced perturbations of host-cell nuclear structures differ considerably among virus families [1], [2]. The viral components in the nuclei are not randomly distributed, but interact with both pre-existing and virus-induced structures and compartmentalized domains [3]–[5].

The nucleus is a highly complex and dynamic organelle that hosts the chromosomes and a large number of proteinaceous nuclear bodies [6]. The chromatin is organized into a decondensed, transcriptionally active euchromatin and more a condensed, generally inactive heterochromatin. Moreover, individual chromosomes reside in distinct nuclear positions, known as chromosome territories [7], [8]. The space between chromosomes, i.e. the interchromosomal domain (ICD), consists of a network of channels initiating at nuclear pores and forming lacunae between the chromosome territories [9]–[11]. Dedicated to specific functions, it harbors non-chromatin nuclear domains, such as transcription factories, splicing speckles, promyelocytic leukaemia bodies and Cajal bodies involved in mRNA transcription, pre-mRNA processing, transcriptional regulation, and processing of nuclear RNA [6], [11]–[14]. The nucleoplasm is a viscous and highly crowded environment surrounding the chromosomes. Nucleoplasmic motility is restricted by its constituents dissolved macromolecules, e.g., proteins, nucleic acids, and sugars [15]–[17]. Also, the chromatin corral comprising DNA condensed with nucleosomal histones H2A, H2B, H3, and H4, restrains the movement of nuclear components by molecular sieving [18], [19]. Molecular interactions of viral proteins with chromatin and nuclear proteins, as well as the supramolecular modification of nuclear architecture, are important determinants of virus infection.

The non-enveloped parvoviruses are among the smallest DNA viruses. Canine parvovirus (CPV) encapsidates its single-stranded negative-sense DNA genome of 5300 bases into an icosahedral capsid of ∼260 Å in diameter [20]. The genome of autonomous parvovirus comprises two transcriptional units, one encoding the capsid proteins VP1 and VP2, and the other the nonstructural proteins NS1 and NS2 [21], [22]. NS1, a nuclear DNA-binding phosphoprotein, has multiple functions in the viral life cycle [23]–[25]. It serves as an initiator and a helicase in viral DNA replication, and as an activator of the viral promoters during diversion of the cellular machinery towards viral protein expression [26], [27]. NS1 has been shown to colocalize with the replicating viral DNA in virus-induced compartments known as autonomous parvovirus-associated replication bodies [28].

An essential cellular replication protein, proliferating cell nuclear antigen (PCNA) encircles the dsDNA and enhances the DNA polymerase delta processivity in eukaryotic replication. PCNA has been shown to localize into the viral replication compartments in baculovirus [29] and Epstein-Barr virus infections [30]. During parvovirus infection, PCNA accumulates in the replication bodies together with the polymerase delta and, is known to be important factor in the viral genome replication in vitro [28], [31], [32].

In this study, we use advanced confocal imaging techniques including photobleaching, fluorescence correlation spectroscopy and photoactivation to clarify the intranuclear processes and molecular interactions in parvovirus infection. The distributions of virus capsids and histone H2B, and dextrans of varying size, were determined so as to understand the size constraints of macromolecular dynamics within the viral replication bodies. Intranuclear diffusion of CPV virus like particles (VLPs) was studied with photoactivable VP2. Moreover, the dynamics and interactions of histone H2B, Enhanced Yellow Fluorescent Protein (EYFP), NS1 and PCNA in infected cells were assessed by quantitative fluorescence recovery after photobleaching (FRAP) and fluorescence fluctuation microscopy (FFM).

## Methods

### Cell lines, Viruses, Constructs and Reagents

Norden laboratory feline kidney (NLFK) cells were grown in Dulbecco's modified Eagle medium (DMEM) supplemented with 10% fetal bovine serum (Gibco, Paisley, UK) at 37°C in the presence of 5% CO_2_. HEK293, HeLa, T98G and TP366 cells were grown as described [33]. For live cell microscopy studies, the cells were seeded on 5 cm glass-bottom culture dishes (1.5 thickness, MatTek Cultureware, Ashland, MA). For FFM imaging and measurements, cells were transfered and transfected on 32 mm cover slips as described in [33]. CPV-2d isolates originated from the infectious plasmid clone (a generous gift from C.R. Parrish, Cornell University, Ithaca, NY [22]). The viruses had been isolated as described in [34]. For infection, the cells were inoculated with CPV (MOI 1–2) and kept at 37°C until live-cell microscopy or fixation.

The plasmids encoding fluorescent proteins, EYFP-PCNA, H2B-EYFP and H2B-ECFP, were generous gifts from Wim Vermeulen (Department of Cell Biology & Genetics, Erasmus MC, Rotterdam, The Netherlands) and J. Langowski (German Cancer Research Center, Heidelberg, Germany). The pEYFP-N3 construct was purchased from Clontech Laboratories Inc. (Mountain View, CA). The plasmid construct, NS1-deYFP is a modification of NS1-EYFP [35]. To prevent production of residual EYFP, the capsid promoter P38 TATA-box sequence was changed with three conserved mutations (tataaat to GatCaaC), and the start codon of EYFP was mutated (Atg to Ctg). The PAGFP-VP2 construct was cloned by replacing the EGFP gene from pEGFP-C1 (Clontech) plasmid with the PAGFP gene [36] to produce the PAGFP-C1 plasmid. The PAGFP plasmid was a generous gift from J. Fransen (NCMLS, Nijmegen, The Netherlands). The CPV VP2 gene was cloned from an infectious plasmid [22] to the 3′- terminus of PAGFP gene flanking the SacI and the KpnI restriction enzyme sites. The correctness of the final product was confirmed by sequencing. Western blot analyzis was used to verify the expression of VP2 in PAGFP-VP2 transfected cells. Here, the cells were transfected with the pPAGFP-VP2 or pEGFP construct, or were infected with CPV. The total cell lysates were analyzed with 10% SDS-PAGE, and were Western blotted with a rabbit antibody (Ab) against VPs [37] or rabbit anti-GFP Ab (Invitrogen Corporation, Carlsbad, CA). Appropriate alkaline-phosphatase conjugated secondary antibodies were used for detection (Bio-Rad, Hercules, CA).

NS1-deYFP and PAGFP-VP2 transfections were performed with TransIT-LT1 reagent (Thermo Fisher Scientific Inc, Waltham, MA) according to the manufacturer's protocol. For studies of protein dynamics during infection, the cells were infected 20–24 h post transfection. NLFK cell lines stably expressing PCNA-EYFP, H2B-EYFP, or H2B-ECFP were established by transfection with an expression vector at 24 h after seeding. After 2 days the DMEM was replaced by DMEM containing 1 mg/ml of geneticin (Sigma Aldrich, St Louis, MO). The cells were then seeded at different intervals until a stable expression was observed by microscopy.

Incorporation of 5-bromo-2-deoxyuridine (BrdU, Sigma) was used to document the DNA synthesis in infected cells. The cells were incubated in DMEM containing 25 µM BrdU for 40 min at 24 h post infection (p.i). Cellular BrdU was detected with a mouse monoclonal Ab (MAb, Santa Cruz Biotechnology, Santa Cruz, CA) followed by Alexa-555-conjugated anti-mouse IgG (Molecular Probes, Eugene, OR). BrdU and PCNA were labeled with anti-BrdU MAb and anti-PCNA (Abcam, Cambridge, UK) Ab, respectively, followed by Alexa-488-conjugated anti-mouse IgG and Alexa-633-conjugated anti-rabbit IgG. Labeling was performed either with or without denaturation of the DNA with 2 M HCl [38]. After labelling the cells were embedded in Mowiol containing Dabco antifade reagent (Sigma). BrdU labeling of chromosomal DNA was achieved by incubating the cells for 24 h with DMEM containing 25 µM BrdU. Unbound BrdU was removed by replacing the medium to DMEM. Cells were infected with CPV, and were fixed with 4% paraformaldehyde (PFA) at 24 h p.i.

CPV was immunostained either with Ab against VPs or with MAb against capsids [39]. Both antibodies were gifts from C.R. Parrish. The bound Abs and MAbs were visualized by Alexa-633 or 555-conjugated anti-mouse IgG, or Alexa-555-conjugated anti-rabbit IgG (Molecular Probes).

### Microinjection

Microinjection of NLFK cells was carried out using a semiautomatic system consisting of a Transjector 5246 and a Micromanipulator 5171 (Eppendorf, Hamburg, Germany) on an Olympus IMT-2 inverted microscope. Needles were pulled from glass capillaries (Clark Electromedical Instruments, Reading, UK) using a P-97 needle puller (Sutter Instruments, Novato, CA). Cultures were grown to 80% confluency on 5 cm glass-bottom dishes. Cells were microinjected with 40 kDa FITC-dextran (2.5 mg/ml), 146 kDa TRITC-dextran (5 mg/ml), or 500 kDa FITC-dextran (2.5 mg/ml). Cells were infected 2 h prior to microinjection and imaged at 20–24 h p.i.

### Timelapse Imaging of Virus Infection

The imaging of chromatin marginalization and the change in nuclear volume was performed with Zeiss CellObserver HS widefield microscope (Zeiss, Göttingen, Germany). The microscope incubator was maintained at 37°C during the imaging process and the CO_2_ concentration was adjusted to 5%. The LD Plan-Neofluar 40× (NA = 0.6) objective was used. A 455 nm LED from a Colibri light source (Zeiss) was used for excitation of the H2B-ECFP and the emitted fluorescence was collected using a 458–502 nm band-pass filter and a Zeiss AxioCam MRm (chip pixel size 6.45 µm). A binning of 2×2 pixels was used to reduce the exposure time below 1 s. Imaging was carried out at 5 min intervals.

### Confocal Imaging

The images for deconvolution were acquired with a laser scanning confocal microscope LSM510 in Axiovert 100 M (Zeiss, Jena, Germany) using a Zeiss Plan-Neofluar 63× (NA = 1.25) oil immersion objective. For replication body imaging, live cells stably expressing H2B-ECFP were transfected with NS1-deYFP, infected and imaged 20–26 h p.i. The stage and the objective were warmed to 37°C before imaging. Image stacks of 30–45 slices were captured with a voxel size of 47 nm in the x and y, and 230 nm in the z (512×512 image, zoom factor 6) dimension. ECFP and EYFP were excited respectively with a 458 nm and a 514 nm laserlines. The ECFP fluorescence was collected using a 475–505 nm band-pass filter and a 530 nm long pass filter for EYFP. The pinhole was adjusted to 1 Airy unit. Capsids were immunolabeled with capsid MAb followed by Alexa-633-conjugated anti-mouse IgG, and their distribution was monitored in cells stably expressing H2B-ECFP. Capsids were detected with a HeNe 633 nm laser and a 650 nm long-pass filter. Imaging parameters used for ECFP were identical to those in replication body studies. The FITC-labeled dextran distribution was imaged using 488 nm excitation and 505–530 nm band-pass filter. The TRITC-labeled dextran was imaged with a 543 nm excitation and the fluorescence was detected with a 560 nm long-pass filter. The voxel size in the dextran imaging experiments was adjusted to 48 nm in the x and y, and to 154 nm in the z dimension. The pinhole was kept at 1 Airy unit. Stack were build-up from 30–55 slices of 512×512 pixel images (zoom factor 6). Multitracking was used to avoid crosstalk.

Imaging of BrdU labelled cells, DNaseI digested cells, and the nuclear volume was preformed with an Olympus FV1000 laser scanning confocal microscope attached to an IX-81 inverted microscope frame (Olympus Tokyo, Japan) with an UPLSAPO 60× (NA = 1.3) objective. For BrdU and DNaseI digestion assay imaging, single-section images were captured with an image size of 512×512 pixels. The pixel size was adjusted to 59 nm and 66 nm for BrdU and DNaseI, respectively. The nucleus size was imaged by capturing stacks of 20–30 images, with a pixel size of 92 nm in the x and y, and 500 nm in the z dimension. The pinhole was set to 1 Airy unit. DAPI and ECFP were excited with the 405 nm laserline, EYFP and Alexa-555 respectively with 515 nm and 543 nm laserlines. The fluorescence was collected respectively with 425–525 nm band-pass, 530–630 nm band-pass and 650 nm long-pass filters.

### FRAP experiments

Various FRAP protocols were used to study the protein dynamics in live cells. Detailed descriptions of the methods employed in this study are provided in supporting information (Text S1).

### FFM experiments

Fluorescence fluctuation microscopy was used to measure the diffusion of EYFP in the nuclei of NLFK cells and of EGFP in various cell lines. Comprehensive description of the method including data analysis is provided in supporting information (Text S1).

### Photoactivable GFP-VP2

Photoactivable (PA) GFP was fused to VP2 for a study of viral capsid protein dynamics in infected and non-infected cells. The Experimental protocol can be found in supporting information (Text S1).

### FRAP data normalization, fitting and Virtual Cell modelling

Various recovery models were used to obtain further information on protein recoveries, see supporting information (Text S1).

## Results

### Capsids and capsid-size dextran have dissimilar intranuclear distributions

**Figure 1:**
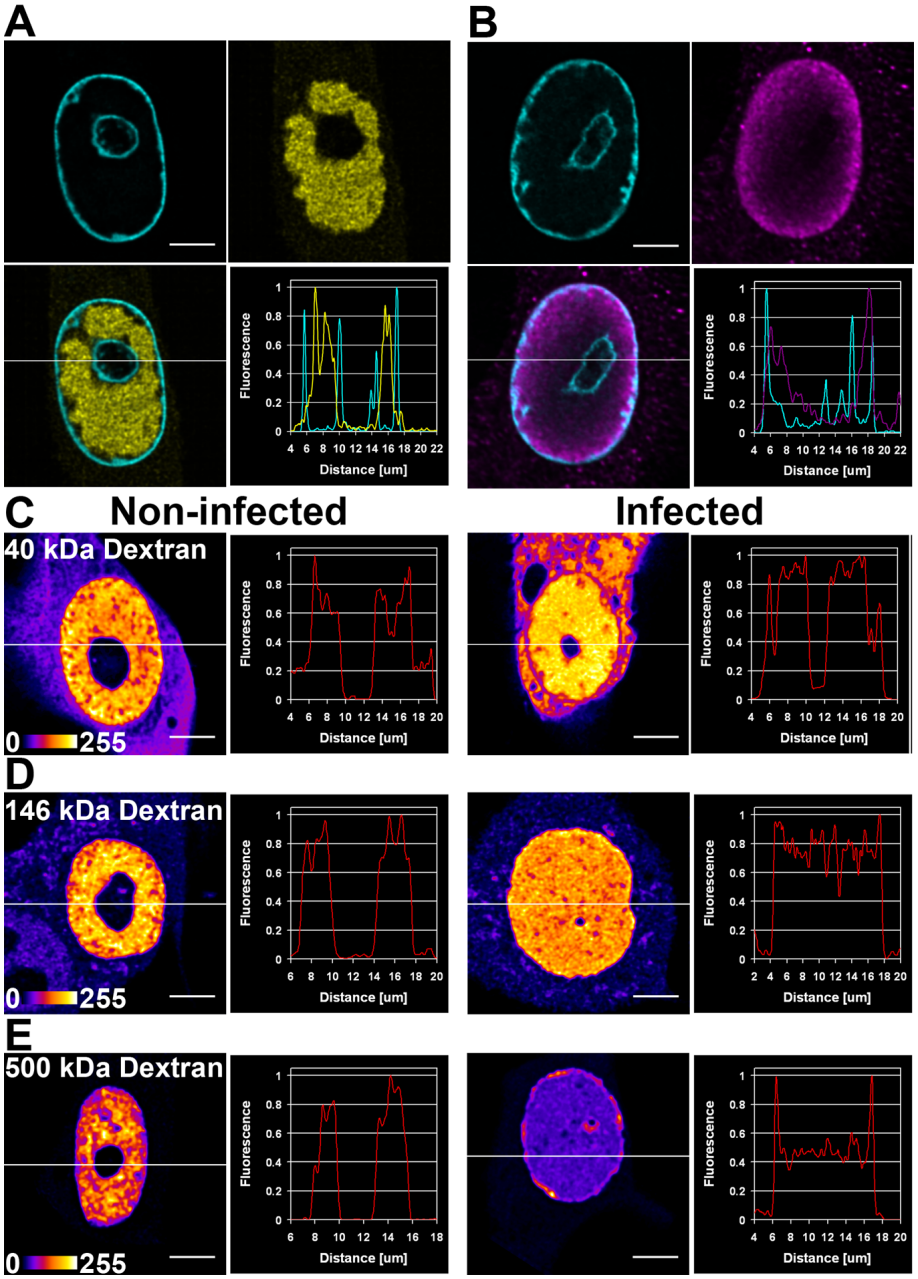
Intranuclear distribution of viral proteins and dextrans. Deconvoluted confocal microscopy images of CPV infected NLFK cells stably expressing H2B-ECFP studied 20–24 h post infection. (A) Live cell images of intranuclear histone H2B-ECFP (cyan) and NS1-deYFP (yellow). (B) Fixed cell images of intranuclear histone H2B-ECFP (cyan) and capsid Ab (magenta). Deconvoluted confocal microscopy images of living NLFK cells showing the distribtions of (C) 40 kDa, (D) 146 kDa and (E) 500 kDa dextrans in a pseudocolor scale. Scale bars, 5 µm.

Our previous studies of CPV infected cells have demonstrated the accumulation of fluorescent NS1 fusion protein into distinct nuclear foci followed by a thorough intranuclear distribution of NS1 [35]. Here we examined the intranuclear localization of fluorescent NS1 in respect to the chromatin distribution, visualized by fluorescent histone H2B-ECFP. Deconvoluted live cell confocal microscopy images showed heterogeneous nuclear distribution of NS1-deYFP at 24 h p.i. (Figure 1A). The chromatin was excluded from the NS1-positive regions, and localized at the nuclear periphery as well as at the nucleoli. In contrast with this, the non-infected control cells displayed a typical chromatin distribution (Figure S6). Deconvolution confocal microscopy of cells fixed at 24 h p.i. revealed a capsid distribution significantly different from that of NS1-deYFP. The capsids were confined to the vicinity of the nuclear membrane in 60±10% (n = 253) of the cells. Analyzis of the line profiles through the nuclear region showed no significant overlap of the distribution of NS1-deYFP with those of histone H2B or the capsids.

To study the accessibility of the nuclear subcompartments, dextrans with a size of 40 kDa (the radius of gyration r_g_≈7 nm), 146 kDa (r_g_≈13 nm) and 500 kDa (r_g_≈24 nm) in size were microinjected to nuclei of infected and non-infected cells. Imaging with a confocal microscope at 20 h post injection showed a homogeneous intranuclear distribution the of the 40 kDa dextran in infected cells (Figure 1C). Similar results were obtained with the 146 kDa dextran, with slightly lower concentration at the nuclear membrane (Figure 1D). The 500 kDa dextran, too, displayed a similarly homogeneous distribution at the replication body area with; however, a pronounced accumulation to the nuclear periphery (Figure 1E). The homogeneous distribution of all the dextrans within the replication bodies indicated lack of compartmentalization in these structures. This was also evident from the line profiles. In the non-infected control cells, all the various sized dextrans had the expected heterogenous distribution. The homogeneous distribution of virus-sized dextran suggested that the virus particles could passively penetrate into the replication body.

### Rapid diffusion of photoactivable virus like particles in replication body

**Figure 2:**
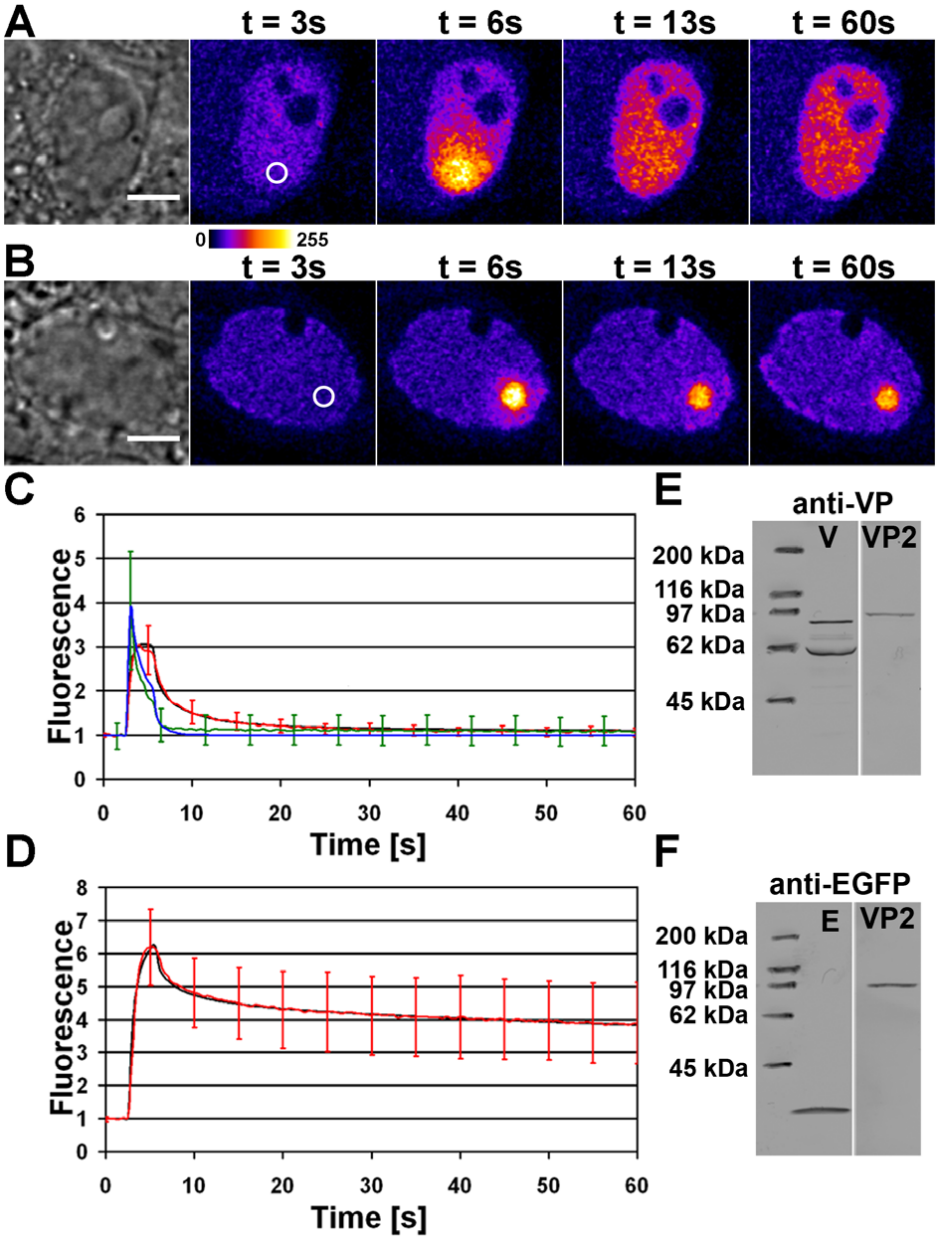
Intranuclear PAGFP-VP2 diffusion dynamics. Confocal microscopy images of PAGFP-VP2 photoactivation studies in (A) non-infected and (B) infected NLFK cell. The activation areas are marked with a white circles. (C) The normalized PAGFP (green) and PAGFP-VP2 (red) fluorescence intensity redistribution in the non-infected cells in addition to PAGFP (blue) and PAGFP-VP2 (black) Virtual Cell simulations of fluorescence redistribution. (D) The normalized fluorescence intensity of PAGFP-VP2 in infected NLFK cells (red) and the Virtual Cell simulation of its redistribution (black). Western-Blot strips of whole-cell lysates of CPV infected (V line) and PAGFP-VP2 (VP2 line) or EGFP (E line) transfected cells were analyzed for fusion protein expression using (E) anti-VP antibodies or (F) anti-EGFP antibodies. Error bars indicate the standard deviation. Scale bars, 5 µm.

The dynamics of capsid protein VP2 was studied in cells expressing this protein fused to a photoactivable GFP (PAGFP). Western blot analyzis confirmed that the PAGFP-VP2 construct had the predicted molecular weight (92 kDa) and was recognized by both the VP antibody and the EGFP antibody (Figure 2E and 2F). In non-infected cells the PAGFP-VP2 proteins were mostly concentrated to the nucleus, in addition to faint cytoplasmic fluorescence. The labelling pattern of VP Ab was similar to the distribution of PAGFP-VP2 while the capsid MAb labels were concentrated in to the nucleus (Figure S2). This indicated that PAGFP-VP2 was able to form VLPs, with a preferential location in the nucleus.

In photoactivation studies the excitation of PAGFP at 488 nm was increased 10–20 fold by an activation laser pulse of 405 nm light. After photoactivation, PAGFP-VP2 diffused rapidly within the nucleus (Figure 2A). The loss of fluorescence on the photoactivation area was simulated by the Virtual Cell software (Figure 2C and S5). Such simulations indicated that, in the activation region of the non-infected cells, immediately after the activation pulse, about 81% of activated PAGFP-VP2 had a diffusion coefficient of 5.0 µm^2^/s, while for about 19% it was 0.02 µm^2^/s. For comparison, simulations of free PAGFP diffusion in non-infected cells showed a much higher diffusion coefficient of 18 µm^2^/s. In infected cells, the majority of PAGFP-VP2 fluorescence remained in the activation region, indicating that either the protein diffusion was slower or that this protein participated in some binding reactions (Figure 2B). The best fit for the data was achieved with Virtual Cell simulations with a two-component system. These simulations indicated that, after the activation pulse, the faster population represented only 26% of the activated PAGFP-VP2 in the activation region with a diffusion coefficient of D = 5 µm^2^/s. The slower population (74%) had a diffusion coefficient of D = 0.001 µm^2^/s (Figure 2D). In these experiments, the PAGFP-VP2 activation region was in the replication compartment and the activated proteins diffused within the replication body. In conclusion, in the infected cells a large fraction of VP2 proteins appeared to be tightly bound. However, our results also indicate that the mobile fraction of PAGFP-VP2 diffuses rapidly both in the viral replication body area and in the nuclei of non-infected cells.

### Virus infection causes chromatin migration to nuclear periphery

**Figure 3:**
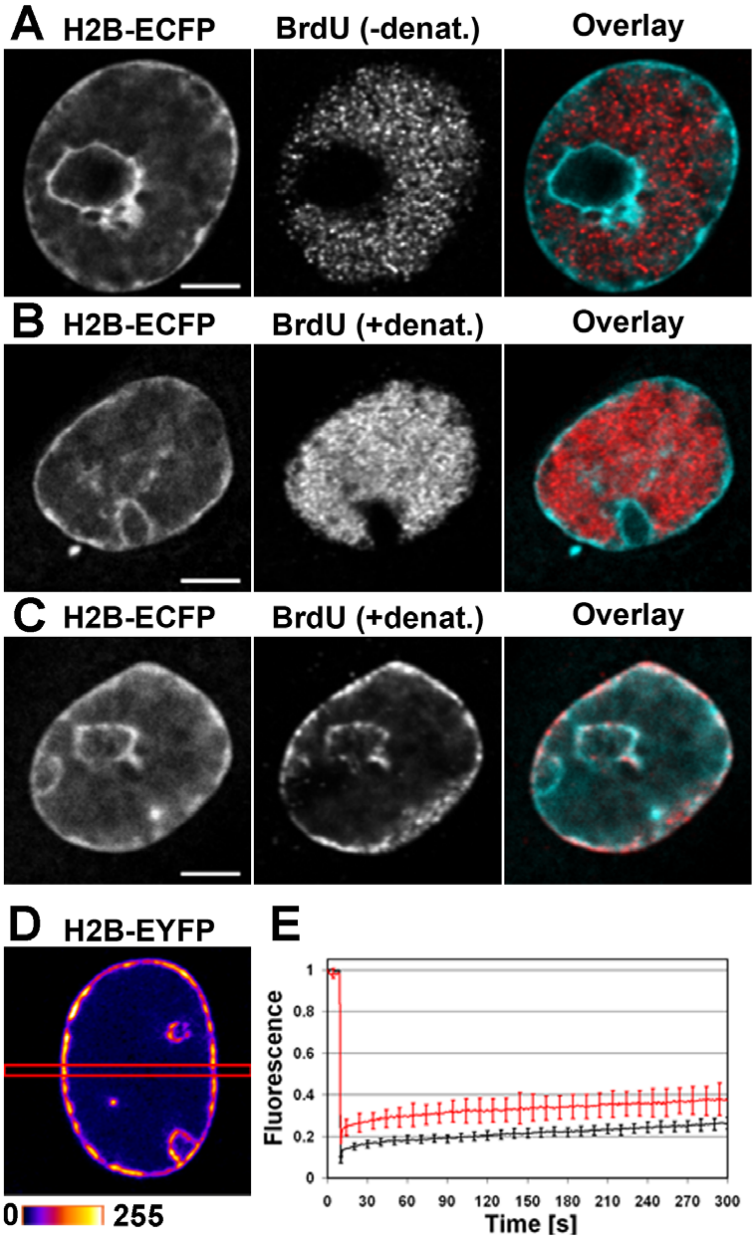
Distribution of histone H2B in NLFK cells. Confocal microscopy images of CPV infected NLFK cells stably expressing H2B-ECFP or H2B-EYFP. Nuclei labelled with BrdU for 40 min at 24 h p.i. The BrdU (red) incorporation was examined (A) without and (B) with denaturation in comparison to H2B-ECFP (cyan). (C) Localization of endogenous DNA, labelled with BrdU prior to infections at 24 h p.i. BrdU (red) distribution in comparison to H2B-ECFP (cyan). (D) Qualitative FRAP analyzis of the H2B-EYFP recovery in the infected NLFK cell stably expressing H2B-EYFP. (E) FRAP recovery curves of H2B-EYFP infected (red) and non-infected (black) NLFK cells. Error bars indicate the standard deviation. Scale bars, 5 µm.

The localization of newly synthesized viral DNA was monitored by BrdU labelling with or without the denaturation step in infected cells stably expressing the chromatin marker H2B-ECFP (Figure 3A and 3B). Confocal imaging of cells at 24 h p.i. revealed incorporation of BrdU in replication bodies of varying size. Interestingly, chromatin was excluded from the regions of newly synthesized viral DNA. In the nucleus with chromatin marginalized to the periphery, the nascent DNA filled the entire nucleus. Fluorescence *in situ-*hybridization at 24 h p.i. showed a similar distribution of viral DNA (Figure S3). To follow the relocalization of endogenous DNA as a result of infection, cells were grown in the presence of BrdU. Prior to the infection, unbound BrdU was removed to avoid its incorporation into the viral DNA. The distributions of BrdU label and H2B-ECFP were identical in the infected cells, indicating that the host cell DNA is marginalized to the nuclear periphery (Figure 3C).

To explore the accessibility of DNA in the replication bodies, DNaseI was applied into detergent permeabilized cells (Figure S1). In the infected, nontreated cells, DAPI labelled both the chromatin ring near the nuclear envelope and the entire replication body. In the infected, DNaseI-treated cells, the replication body was no longer visible. Most importantly, in these cells neither NS1-deYFP nor PCNA-EYFP was detectable any longer in the nucleus. These results suggest that the viral DNA is involved in the binding of NS1-deYFP and PCNA-EYFP in the replication bodies.

Time-lapse analyzes were performed to analyze chromatin marginalization in the infected cells stably expressing H2B-ECFP. Imaging revealed a rapid enlargement of the ICD at 16–24 h p.i. (Movie S1).

FRAP experiments were performed on cells stably expressing H2B-EYFP to assess virus-infection-induced alterations in H2B binding. The fluorescence recovery of a bleached rectangular area (1 µm in width) was followed for 5 minutes. Interestingly, the recovery of H2B-EYFP fluorescence was found to be as slow in infected as in non-infected cells even though the corresponding distributions of H2B-EYFP were drastically different (Figure 3D and 3E).

### Amount of nuclear DNA and nuclear volume increase in infected cells

**Figure 4:**
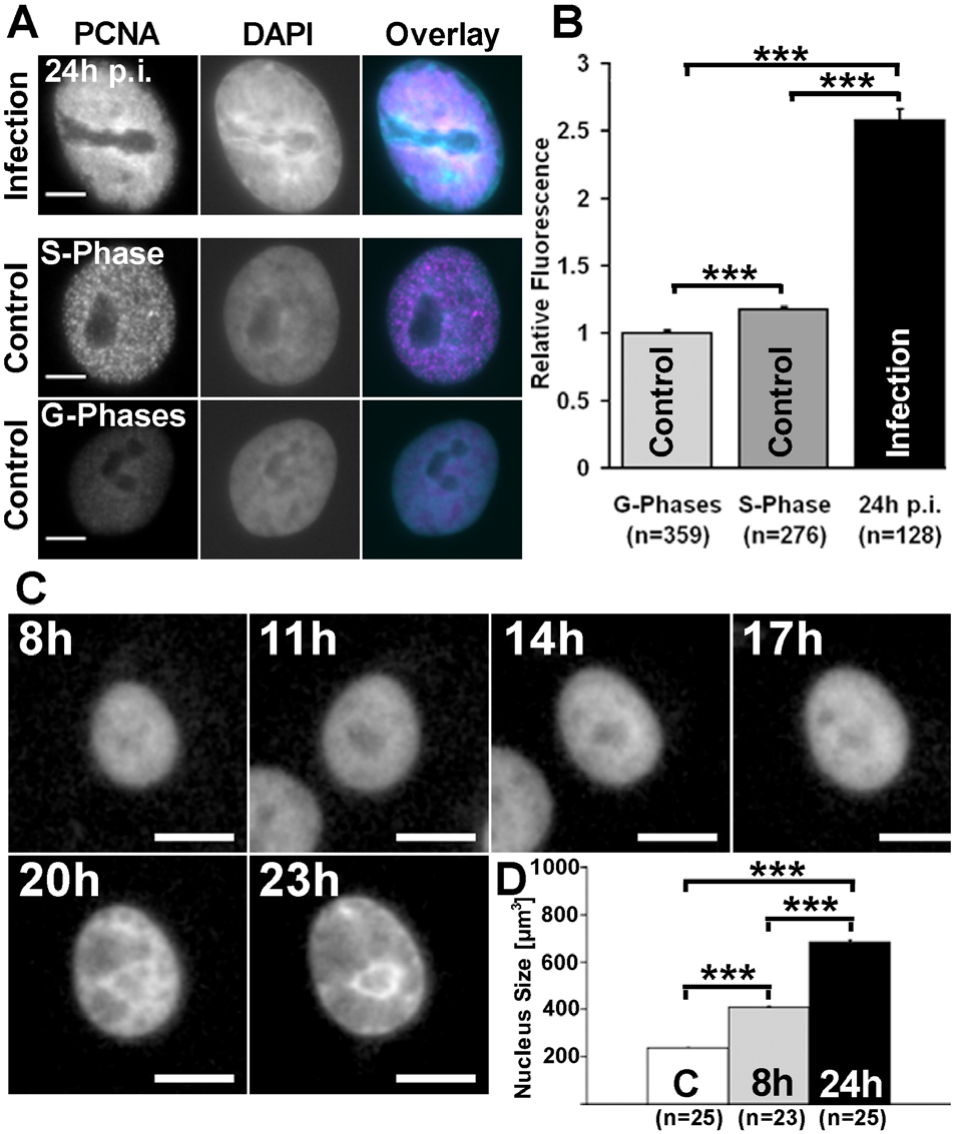
DNA content and nuclear size in infected and non-infected cells. Widefield microscopy images of NLFK cells. (A) Infected and non-infected G1/G2 and S phase cells labelled with anti-PCNA (red) antibody and DAPI (cyan). (B) DAPI fluorescence intensity measured from G phase, S phase and infected cells. (C) Timelapse imaging of infected H2B-ECFP expressing cells showing an increase in the nuclear size. (D) Nuclear volumes from fixed H2B-EYFP expressing cells. Error bars indicate the standard error of the mean. Confidence interval p<0.001 is marked with ***. Scale bars, 5 µm.

The above experiments showed that chromatin-depleted replication bodies were relatively homogeneous in structure, accessible to virus-size particles, and sensitive to DNaseI. Next, we measured the relative amount of DNA and the nuclear volume in infected cells. The DAPI labelling indicated that the DNA content was 2.5 times higher in infected than in non-infected cells (Figure 4A and 4B). In the control cells, different cell cycle phases were separated by PCNA labeling. In the G1/G2 cell cycle phases PCNA showed a homogeneous distribution, whereas in the S phase it displayed a highly punctuate pattern [40].

Timelapse imaging of the infected cells stably expressing H2B-ECFP showed an increase in the nuclear volume (Figure 4C). In cells fixed at 8 h p.i. the increase was 1.7 fold in comparison with non-infected cells. 24 h p.i. the increase was 2.9 fold (Figure 4D). As both the nuclear volume and the amount of DNA increased, we propose that the DNA concentration remained relatively unaltered.

### Protein mobility is increased in viral infection

**Figure 5:**
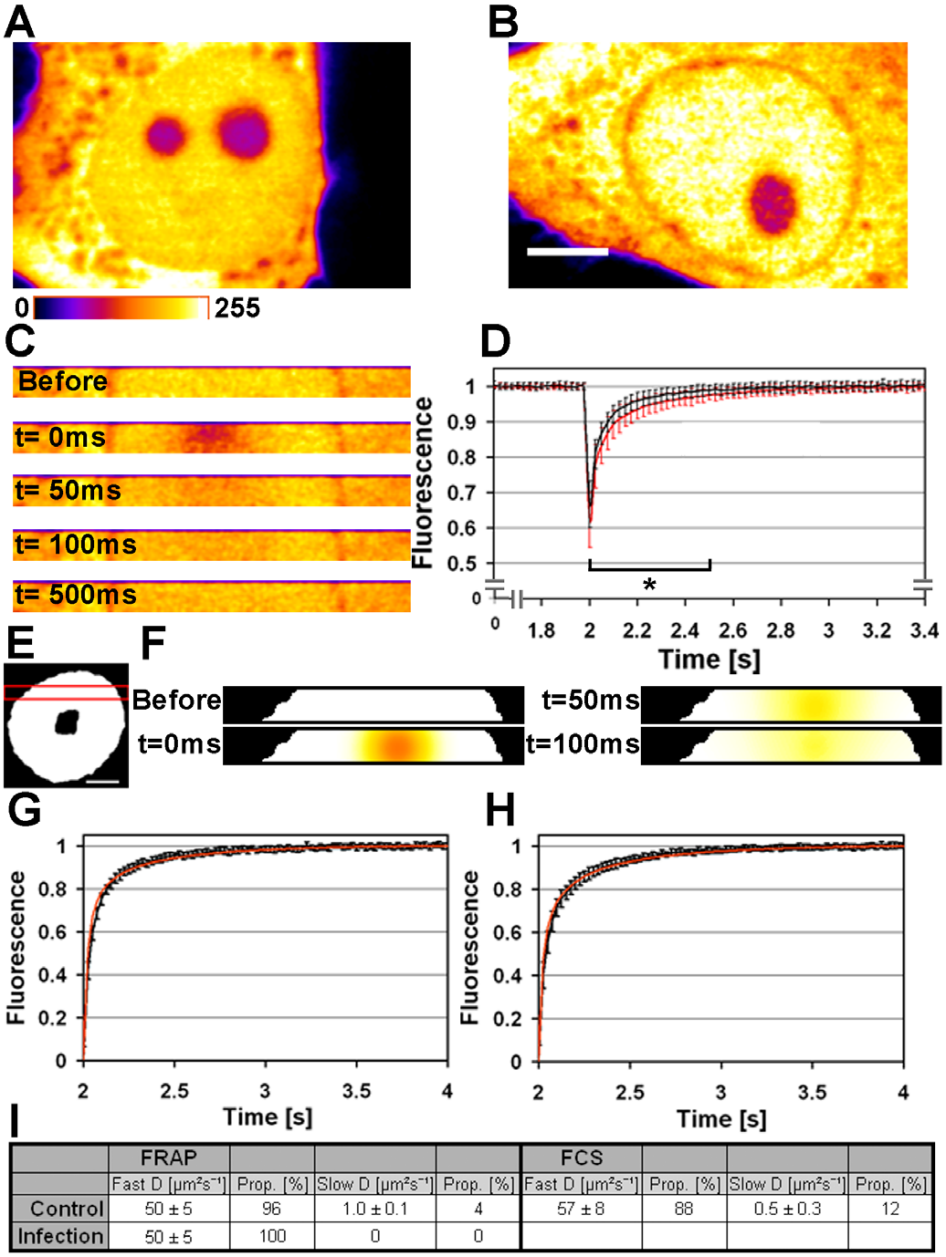
EYFP diffusion in nuclei of infected and non-infected cells. FRAP experiments and Virtual Cell Simulations of EYFP diffusion. (A) Non-infected cell with a homogeneous intranuclear distribution of EYFP. (B) Infected cell showing a uniform distribution of EYFP in the replication body, with a darker rim visible near the nuclear membrane. (C) FRAP experiments of infected cells performed with a high frame rate to capture the rapid fluorescence recovery. (D) Fluorescence recovery curves showing a faster recovery in the infected (black) than in the non-infected (red) cells. (E) Nuclear geometry in the simulated EYFP FRAP experiment. (F) Simulated FRAP recovery in non-infected cells. Measured recovery (black) in infected cell (G) and in non-infected cell nuclei (H) in comparison to the simulated experiment (red). (I) Summary of the results obtained with FRAP and FFM. Confidence interval p<0.05 is marked with *. Error bars indicate the standard deviation. Scale bars, 5 µm.

The above experiments indicated that in infected cells the nuclei are drastically reorganized as the replication bodies form. Next we studied if the intranuclear diffusion of proteins is affected by the virus infection. Subsequently, the general protein diffusion was analyzed by quantitative FRAP, FFM and Virtual Cell simulations of infected and non-infected cells expressing free EYFP. Confocal microscopy imaging revealed a homogeneous distribution of EYFP throughout the non-infected cells (Figure 5A). In comparison, in the infected cells, an otherwise homogeneous distribution of EYFP was accompanied by a darker rim close to the nuclear membrane indicating a decrease in the concentration of EYFP in this region (Figure 5B). This area is packed with chromatin (Figure S6), suggesting that it is the chromatin that hinders the EYFP diffusion into this region. In addition, the intranuclear recovery of the EYFP signal was extremely fast (Figure 5C and 5D). The data were normalized, averaged and fitted by a free diffusion model. The free diffusion coefficient of EYFP in the non-infected control cells was D = 19±2 µm^2^/s. At an early phase of recovery the fit showed small inconsistency, whereas the fit for FRAP data measured from infected cells was better, yielding a diffusion coefficient of D = 28±3 µm^2^/s (results not shown). However, the model used for fitting of the FRAP data does not take into account the diffusion during the bleach phase, which can lead to ∼4–5 times underestimated diffusion coefficients [41]. In addition, it assumes that the diffusing molecules are of a single population. In order to clarify these issues, FFM measurements were conducted to study EYFP diffusion in non-infected control cells. The FFM results indicated that the observed EYFP diffusion could be explained with a two components system, in which 88% of EYFP has a diffusion coefficient of D = 57±8 µm^2^/s, and the rest of D = 0.5±0.3 µm^2^/s. Notably, very similar diffusion coefficients and population distributions were obtained from nuclei of HEK293, HeLa, T98G and TP366 cell lines (Figure 5I and S7). Accordingly, we constructed a two-component Virtual Cell model, which allowed us to simulate the FRAP experiments (Figure 5E, 5F and S4). The fluorescence recovery measured from the control cells, could now be reproduced by a simulation, in which 96% of EYFP had a diffusion coefficient of D = 50±5 µm^2^/s, and 4% of D = 1±0.1 µm^2^/s (Figure 5H). In infected cells, the slowly diffusing population of EYFP had disappeared, and corresponding simulations with a single component system indicated an EYFP diffusion coefficient of D = 50±5 µm^2^/s (Figure 5G). These results show that in the infected cells the nuclear mobility of proteins is increased.

### NS1-deYFP recovery can be explained by two different models

**Figure 6:**
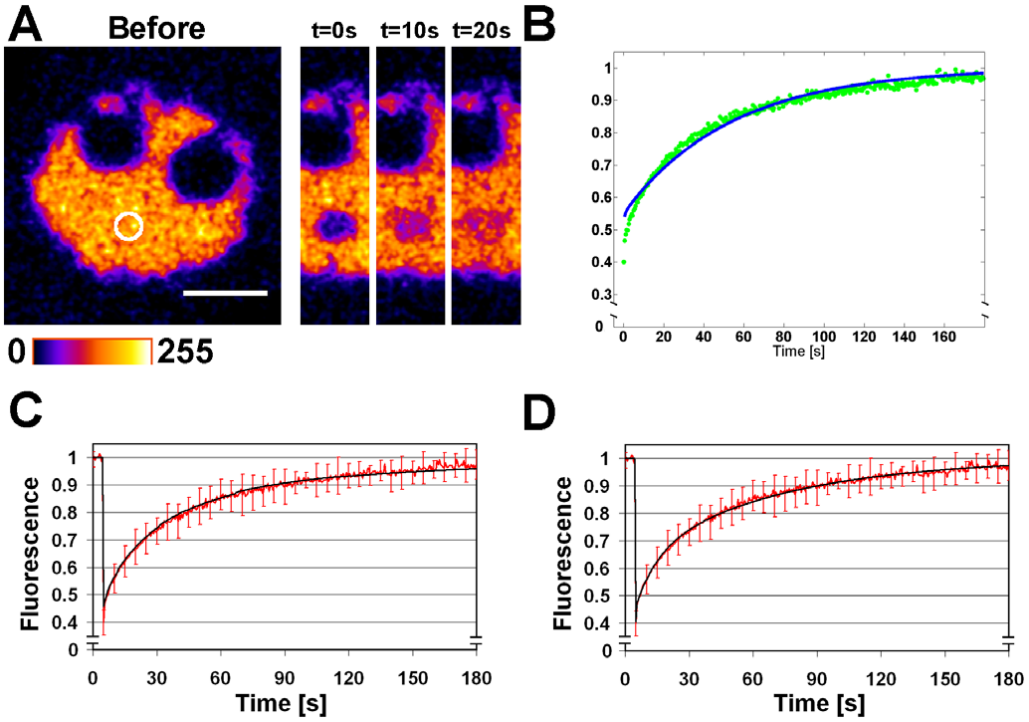
Intranuclear NS1-deYFP dynamics. Infected NLFK cell expressing NS1-deYFP. (A) NS1-deYFP distribution shown in a pseudocolour scale. (B) NS1-deYFP fluorescence recovery (green) and a fit by the full model (blue). (C) Virtual Cell model result (black) for the NS1-deYFP recovery (red) with a mobile NS1-deYFP binding partner. (D) Virtual Cell model result (black) for the NS1-deYFP (red) recovery with two distinct binding sites with different affinities for NS1-deYFP. Error bars indicate the standard deviation. Scale bar, 5 µm.

Next we analyzed protein dynamics in the replication structures. NS1-deYFP binding dynamics were studied by quantitative FRAP and mathematical modelling of the recovery data. A small circular area in the middle of the nucleus was bleached, and the fluorescence recovery was measured at 0.5 s intervals (Figure 6A). The FRAP data were normalized, averaged and fitted by models of Sprague et al. [42]. Data fitting with the binding-dominated recovery model gave poor results (data not shown). The data were then fitted by the full model that includes diffusion of free molecules. Even though the results of the fit were better (Figure 6B), the diffusion coefficient of the free NS1-deYFP was unexpectedly small (D = 1.78 µm^2^/s). A simple mass scaling of the EYFP diffusion coefficient (D = 28±4 µm^2^/s) indicated that the NS1-deYFP diffusion coefficient is D = 18±3 µm^2^/s in infected cells. Using this diffusion coefficient gave again poor fit results (data not shown). This indicated that the models used did not explain the NS1-deYFP recovery. Both models assumed an immobile binding partner and only one binding reaction. Therefore we used two other recovery models: binding of NS1-deYFP at a mobile site, and at multiple immobile sites. The mobile-binding-site hypothesis was tested with the Virtual Cell software. The diffusion coefficient of the genome was approximated as D = 0.01 µm^2^/s (see the Discussion section) and the NS1-deYFP diffusion coefficient was set to18 µm^2^/s. The fit of our data with the Virtual Cell model was good, suggesting that the binding partner of NS1-deYFP was mobile (Figure 6C). The resulting pseudo on rate k_on_^*^ and off rate k_off_ of NS1-deYFP yielded a binding time of 250 s (k_off_ = 0.004±0.0007 s^−1^) and a free diffusion time of 42 s (k_on_^*^ = 0.024±0.004 s^−1^), respectively. Based on these rates, we can conclude that the majority (86%) of NS1-deYFP is bound and only a small fraction (14%) is freely diffusing.

The multiple-binding-site hypothesis was tested with the Virtual Cell software, assigning to NS1-deYFP two distinct and immobile binding sites with different affinities. We obtained again good fit results (Figure 6D). The first site has a high affinity, and with a binding time of 83 s (k_off_ = 0.012±0.002 s^−1^). The second site has a low affinity, and with an average binding time of 10 s (k_off_ = 0.10±0.02 s^−1^). The free diffusion time of NS1-deYFP was 8.1 s (for high affinity site k_on_ = 0.024±0.004 s^−1^, and for low affinity site k_on_ = 0.10±0.02 s^−1^). According to this model, at equilibrium 25% of NS1-deYFP was bound to low affinity sites, 50% to high affinity sites, and 25% was freely diffusing. Both of the Virtual Cell models indicated that the binding of NS1-deYFP is very strong, and the binding times are in range of tens of seconds.

### PCNA-EYFP is strongly associated with replication bodies

**Figure 7:**
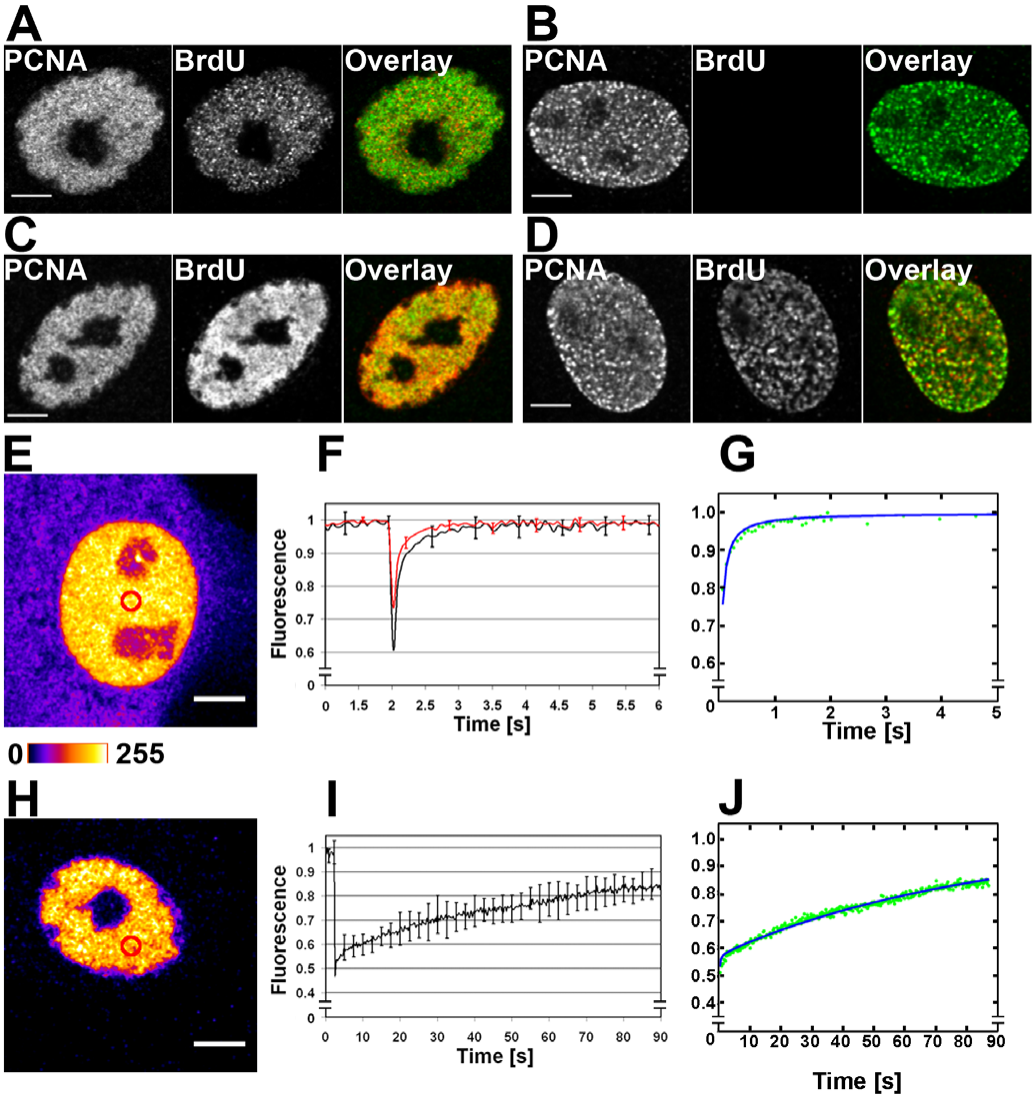
PCNA-EYFP dynamics. BrdU (red) and PCNA (green) labels in NLFK cells with or without a denaturation step. (A) BrdU positive small foci in the PCNA labelled replication body of an infected cell observed without DNA denaturation. (B) Distributions of BrdU and PCNA in the S phase of a non-infected cell without DNA denaturation. (C) BrdU and PCNA labelled replication body in an infected cell with DNA denaturation (D) BrdU and PCNA in a non-infected cell with DNA denaturation. FRAP experiments were performed in cells stably expressing PCNA-EYFP. (E) A G phase cell with a homogeneous intranuclear distribution of PCNA-EYFP shown in a pseudocolour scale. (F) FRAP recovery of PCNA-EYFP (black), and EYFP (red) used as a control. (G) Recovery of PCNA-EYFP (green) fitted by the free diffusion model (blue). (H) An infected cell with PCNA-EYFP concentrated into the viral replication body. (I) Recovery of PCNA-EYFP in infected cells. (J) Recovery data (green) fitted by the full model (black). Error bars indicate the standard deviation. Scale bars, 5 µm.

PCNA is an essential protein in parvovirus genome replication [31]. We thus studied first the spatial interaction of PCNA with nascent viral DNA in BrdU labelled cells. At 24 h p.i., PCNA accumulated in the replication bodies and the intranuclear BrdU was found in 560±40 small intranuclear foci (Figure 7A). In contrast with the non-infected cells (Figure 7B and 7D), these foci were detected without denaturation of the DNA suggesting the presence of nascent ssDNA. In infected cells studied after denaturation BrdU was homogeneously distributed in the replication body area (Figure 7C).

The PCNA-EYFP dynamics in the replication bodies was then studied by FRAP. In non-infected cells the PCNA-EYFP distribution was homogeneous (Figure 7E). The diffusion coefficient of EYFP in similar experimental conditions was D = 15±3 µm^2^/s. Based on this, simple mass scaling gave a diffusion coefficient of D = 12±2 µm^2^/s for the PCNA-EYFP monomers, D = 8±2 µm^2^/s for trimers and D = 7±1 µm^2^/s for dual trimers. The recovery of PCNA-EYFP in non-infected cells was fast (Figure 7F), and fitting by the free diffusion model resulted in a diffusion coefficient of D = 9±2 µm^2^/s (Figure 6G). This value is in good agreement with the diffusion coefficient estimated for the trimer (see the Discussion section). In comparison, the recovery of PCNA-EYFP in the infected cells was drastically slower (Figure 7H and 7I). The recovery data were fitted by the full model with a free diffusion coefficient of 9±2 µm^2^/s. The fit yielded binding time and a free diffusion time of 83 s (k_off_ = 0.012±0.002 s^−1^) and 111 s (k_on_^*^ = 0.009±0.002 s^−1^), respectively (Figure 7J). Of note, the binding time of PCNA-EYFP was close to that of NS1-deYFP.

## Discussion

Exploitation of the cellular nuclear replication machinery by several DNA viruses is accompanied by alterations in the nuclear architecture and dynamics. Although the assembly steps of some DNA viruses are relatively well known, the intranuclear dynamics of replication structures are poorly understood. For parvoviruses the replication processes at the molecular level are relatively well established [26], [27], [43], and the formation of nuclear replication bodies at late stages of infection has been demonstrated [28], [35], [44]. Here we have explored the changes in intranuclear architecture triggered by a parvovirus infection and dynamics of viral replication bodies.

Late atfter CPV infection, the fluorescent NS1-containing replication body filled the entire nucleus except the nucleolus, and chromatin was confined to the vicinity of the nuclear envelope. Also the viral capsids tended to accumulate close to the nuclear membrane. These results are consistent with observations made previously on herpesviruses and baculoviruses, showing that the RNA transcription, the DNA replication and the virion assembly take place in distinct locations [45], [46]. Our data suggest that either the capsids are assembled in the vicinity of the nuclear membrane or that they are too large to enter the replication body. On the other hand, nuclear export of the assembled capsids might be their transport-limiting factor, thus causing their accumulation at the nuclear periphery.

To examine whether it is the size constraint that explains the periferal localization of the capsids, we microinjected dextrans of various sizes to the nuclei of infected cells. We found that none of the dextrans were completely excluded from the replication compartment. It has been reported previously that the nucleoplasm is not fully accessible to dextrans; the dense chromatin regions exclude dextrans of >70 kDa [47], [48]. However, our results indicate that the viral replication body is accessible to dextrans of even 146 kDa, which to a large extent are repelled from the condensed chromatin domains. This suggests that within the host-cell nucleus the viral DNA is mostly uncondensed.

It is the radius of gyration of a particle that defines the size of the mesh pore through which it can enter. The dextran particles of 40 kDa, 146 kDa and 500 kDa have gyration radii of r_g_≈7 nm, r_g_≈13 nm and r_g_≈24 nm, respectively. For spherical proteins, the relation of particle radius (r) to the radius of gyration is *r = r_g_/0.775* [47], whereby the above radii of gyration correspond to protein radii of r≈9 nm, r≈17 nm and r≈31 nm, respectively. Our results show that particles with the size of a CPV capsid (r = 13 nm) are able to spread passively throughout the replication body. Taken together, these results suggest that, whilst the capsids – in terms of their size – are able to enter the structurally non-compartmentalized viral replication body, other factors induce their localization into close proximity of the nuclear envelope.

Observations on the structure of the replication body raised the question of capsid protein dynamics. The diffusion dynamics were examined using photoactivable GFP (PAGFP) fused N-terminally with the major capsid protein VP2. It is known that such a fusion allows for assembly of VLPs [49]. Also, in the case of another parvovirus, the minute virus of mice, the capsid proteins enter the nuclei only in trimeric form [50]. Our analyzis of non-infected cells showed accumulation of the PAGFP-VP2 into the cell nuclei, in which it was also recognized by the anticapsid antibody. Simulations of the photoactivation experiments suggested the existence of two separate PAGFP-VP2 complexes in the activation region, 81% with diffusion coefficient of 5 µm^2^/s and the remaining 19% of 0.02 µm^2^/s. Free PAGFP redistributed rapidly after activation. A Virtual Cell simulation indicated a diffusion coefficient of D = 18 µm^2^/s. Consequently, mass scaling gave theoretical diffusion coefficients of 11.9 µm^2^/s for the PAGFP-VP2 monomer, 8.2 µm^2^/s for the PAGFP-VP2 trimer, and 3.0 µm^2^/s for the entire capsid. Previously, a diffusion coefficient of 17 µm^2^/s has been measured with fluorescence correlation spectroscopy (FCS) for CPV VLPs in buffer [51]. Based on the observation that the diffusion coefficients of virus-sized dextrans are ∼25% smaller in the nucleus than in water [52], the nuclear diffusion coefficient of the CPV VLP can be estimated to be D = 4.3 µm^2^/s. This, together with prior results on VLP assembly [51], trimer nuclear import [50], and our immunofluorescence data, suggests that the faster component corresponds to freely diffusing VLPs. An adeno associated virus, another parvovirus, has been shown to move along linear tracks in the nucleus [53]. Our data suggest that the motion of CPV capsids within the nucleus is not active but occurs by passive diffusion. Moreover, in infected cells the faster capsid population was shown to diffuse with the same diffusion coefficient as in the control cells. This allows for the capsids to travel a distance of 10 µm in 3.3 seconds. Virtual Cell simulations indicated that 74% of PAGFP-VP2 redistributed extremely slowly from the activation region with a diffusion coefficient of 0.001 µm^2^/s, with the activation spot still visible ten minutes after activation. This fraction is likely to be bound to DNA as shown previously for the LuIII parvovirus [54]. However, the proportions of rapidly and slowly diffusing populations are directly related to the photoactivation of these species and do not necessarily represent the steady state conditions in the nuclei. When analyzing the entire nucleus, 98% of the activated PAGFP-VP2 molecules were rapidly diffusing in the non-infected cells, by comparison with 80% in the infected cells.

The highly organized chromatin occupies a large proportion of the nucleus and controls the mobility of nuclear bodies [19], [55]. We found that enlargement of the replication body was concomitantly followed by marginalization of the chromatin to the nuclear periphery. In addition, the marginalized chromatin restrained EYFP diffusion to this region. This indicates that the chromatin is in a highly condensed state, since it has been reported that proteins of even ∼1 MDa can fully access the chromatin [18]. The volume previously occupied by chromatin was filled by the newly synthesized viral DNA. Furthermore, this process of virus-infection-induced marginalization was fast, occurring in hours. These findings are compatible with previous observations on the perinuclear marginalization of chromatin due to some dsDNA viruses, herpesviruses and baculoviruses [56], [57].

Within the nucleus, the histone H3 protein has been shown to associate with the DNA of herpes simplex virus after its release from the virion, but not with the newly replicated viral genome [58]. Similarly, our results on chromatin marginalization suggest that the nucleosomes are not assembled at the newly synthesized CPV DNA. Moreover, our FRAP data indicate that infection does not affect the slow chromosomal binding of H2B-EYFP.

The intranuclear dynamics of proteins and inert particles such as EGFP or fluorescent dextrans have previously been studied by FCS [33], [59], single particle tracking [60] and FRAP [52]. The diffusion of proteins is more complex in the nucleus than in the cytoplasm. Protein concentration inside the nucleus is approximately 10% [61], and chromatin occupies 5–12% of the nuclear volume [62]. This high macromolecule content could lead to molecular crowding and hindered diffusion [63]. The recent study on free diffusion of EGFP monomers – tetramers in the living mammalian cell nuclei revealed a biphasic system for EGFP diffusion [33]. In this system a portion of EGFPs diffused freely, in addition to a portion with a significantly slower diffusion. Surprisingly, it was reported that neither the diffusion coefficient, relative amount of slowly diffusing EGFP, nor their diffusion coefficients, were dependent on the chromatin density.

The previous studies of nuclear diffusion have been carried out on non-infected interphase cells, whereas we examined the effects of virus infection on protein diffusion. FRAP and FFM studies revealed a two-component system for EYFP diffusion in non-infected NLFK cells, with the diffusion coefficient for faster-component of D = 50±5 µm^2^/s and D = 57±8 µm^2^/s. These data are in good agreement with those reported recently for EGFP inside the cell nuclei [33]. In addition, the FFM results from 5 different cell lines indicated that a considerable portion of the fluorescent proteins showed slower nuclear mobility in comparison to the freely diffusing population. However, the slower population disappeared in the infected cells, indicating unconstrained diffusion for EYFP. As the average protein size in mammalian cells is 53 kDa [17] we suggest that the observed the mobility increase of EYFP (molecular mass of 26 kDa) reflects a general increase in protein mobility in infected cells. According to the Smoluchowski relation, the maximum rate of binding between two interacting species is directly related to their diffusion [64] and consequently to their encounter probability. With an increased mobility in the infected cells, the protein binding reactions are expected to be faster. An enhanced kinetics of replication and assembly would be of obvious benefit to the virus. Higher molecular crowding has been shown to raise the DNA melting temperature and thereby to enhance the rate of hybridization [61]. Likewise, lower molecular crowding decreases the DNA hybridization affinity. This might help to maintain the replicated CPV in single-stranded form prior to assembly. Moreover, conditions of lower molecular crowding favour binding of the single strand binding protein RPA [65] to ssDNA, thus preventing DNA hybridization.

**Figure 8:**
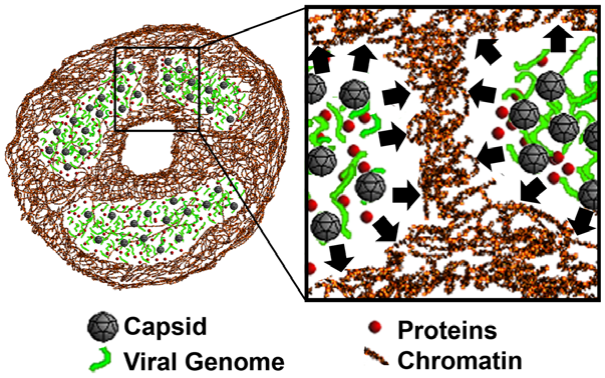
Schematic representation of the replication body enlargement. The proteins, viral DNA, and capsids accumulated into the replication body. The viral DNA has a loose conformation and does not hinder the diffusion of proteins. The replication body components continuously collide with the chromatin causing thereby an osmotic pressure (black arrows) leading to chromatin marginalization.

The depletion attraction phenomenon has been suggested to be involved in vesicle clustering and in the formation of nuclear bodies [66]. According to this theory, macromolecular complexes “feel” an osmotic pressure arising from a continuous stream of collisions with smaller molecules. Due to geometrical constraints, this pressure can be unevenly distributed leading to an attractive force pushing large complexes together. In view of our results for histone relocalization, dextran, and newly synthesized DNA distribution, we propose a model in which chromatin marginalization is induced by depletion attraction caused by enlargement of the viral replication compartment (Figure 8). The viral genomes and proteins exert an osmotic pressure on the chromatin, which leads to enlargement of the ICD and finally to the exclusive chromatin marginalization. However, the loose structure of the replication body still allows for efficient protein diffusion.

It has been suggested that the parvoviral NS1 protein shows non-specific DNA binding *in vivo* in the absence of virus infection [35]. However, our FRAP data indicating long binding times of NS1 inside the replication body were not well fitted by the recovery models of Spraque et al. [35], [42], which assume the binding partner of fluorescent NS1 to be immobile. To further analyze the FRAP data, we used the Virtual Cell software in simulation of NS1 dynamics. In these analyses, the bleach pulse, bleaching caused by the imaging, and the time lag between the bleach and taking the first frame, were taken into account. The FRAP recovery of NS1-deYFP could be explained by two separate Virtual Cell models. In the first model the binding partner of NS1-deYFP was assumed to be mobile and to diffuse slower than mRNA, with a diffusion coefficient of D = 0.01 µm^2^/s [67]. We hypothesized that the mobile binding partner is the viral genome. However, motility of the viral genome is improbable, as earlier studies have indicated that exogenous DNA in the cell nucleus is essentially immobile [68]. The Minute virus of mice genome has been shown to contain multiple copies of two distinct binding sites for NS1 [69]. Based on this and the Virtual Cell simulations with a mobile genome, a second model with two discrete binding sites was considered. This model gave an excellent fit to our data. The longer binding time (83 s) could reflect the time of viral genome synthesis, since NS1 functions as a helicase in the viral DNA replication. A similar binding time was measured for PCNA-EYFP, another component participating in the genome replication.

PCNA is among the most important proteins in viral DNA replication, and has been found to accumulate in the parvoviral replication body [28], [35]. The nuclear distribution of PCNA is cell-cycle dependent; in the S phase it is concentrated in the DNA replication foci, while in the G_1_/G_2_ phase its distribution is homogeneous [40]. Our FRAP experiments on PCNA-EYFP dynamics performed in non-infected cells indicated free diffusion with a diffusion coefficient of 9±2 µm^2^/s. This is compatible with the theoretical calculated diffusion coefficient of 8±2 µm^2^/s for a PCNA-EYFP trimer. Cellular PCNA has been reported to form homotrimers and possibly loose double trimers [70]. Notice that, the diffusion coefficient of PCNA-EYFP is slightly smaller than the reported EGFP-PCNA effective diffusion coefficient, 15 µm^2^/s. The small difference in the results may be due to differences in modeling. In the S phase PCNA associates strongly to the replication foci with reported residence times ranging from ∼25 s half-life [71] to a negligible turnover, indicative of a long half-life [40]. Our data suggest that in the G_1_/G_2_-phase PCNA-EYFP diffuses freely as a trimer. However, in the infected cells PCNA-EYFP recovered slowly, with a binding time of 83 s. Similar binding times have been reported for many transcription related or chromatin binding proteins (binding times ∼3–180 s) with the exception of H2B (binding time >3600 s) [72]. It is known that parvoviruses which can control their genome packaging sense, produce predominantly single ssDNA genomes [73]. As PCNA is thought to remain bound to the DNA strand as long as the polymerase proceeds along its template, and since identical binding time was measured for NS1, we propose that the binding time of 83 s corresponds to the viral genome replication time. With a single stranded viral genome of 5300 bases, this would correspond to a synthesis rate of 64 bases/s - approximately twice that of cellular double-stranded DNA, 33 base pairs/s [74], but in the range of Epstein-Barr virus synthesis rate, 5–78 base pairs/s [75]. Even faster DNA replication has been reported for adenovirus infection, with a seven times higher DNA replication activity than in non-infected cells [76].

The binding times measured for PCNA-EYFP and NS1-deYFP were identical, even though the recoveries of these proteins were fitted by different models. These findings imply that both PCNA and NS1 stay bound to the viral genome during replication, thus supporting the parvoviral genome replication model as proposed in [43].

Our results provide a comprehensive description of the parvovirus-infection-induced modifications in the nucleus. The parvoviral replication body is a complex structure that alters the binding properties of endogenous proteins, displaces the host DNA and modifies the nuclear microenvironment in a way that leads to increased protein mobility. The change in protein mobility can favour the viral replication by enhancing the rate of binding reactions and by reducing the likelihood of ssDNA hybridization.

## Supporting Information

Movie S1 Stably ECFP-H2B expressing cells were infected and imaged from 5 h p.i. to 24 h p.i. At 16–24 h p.i. clear increase in interchromosomal space was evident.
(10.48 MB MPG)

Figure S1 Effect of DNaseI treatment on non-infected and infected NLFK cells. Fixed cell confocal microscopy images of dsDNA (DAPI label), chromatin (H2B-EYFP), NS1-deYFP and PCNA-EYFP after permeabilization and treatment with buffer or DNaseI. Unapparent nuclei are encircled. Scale bars, 5 µm.
(0.53 MB TIF)

Figure S2 Intracellular localization of capsids and viral VP proteins in PAGFP-VP2 expressing cells. NLFK cells expressing PAGFP-VP2 were immunolabelled with antibodies that recognize intact capsids or VP proteins. Distribution of PAGFP-VP2 (green) together with (A) capsid MAb (red) or (B) VP Ab (red). Secondary antibodies used were Alexa-555-labled anti-mouse IgG and anti-rabbit IgG. Scale bars, 5 µm.
(1.10 MB TIF)

Figure S3 Fluorescence in situ labelling of viral genome. Infected cells were labelled with CPV genome specific FISH probe at 24 h p.i. The FISH probe labelled the viral replication compartment inside the nucleus. Scale bar, 5 µm.
(0.54 MB TIF)

Figure S4 Schematic presentation of NS1-deYFP Virtual Cell model. Schematic representation of the Virtual Cell simulation showing the molecular species (green) and reactions between them (yellow). In the model, fluorescent NS1-deYFP (NS1) reacts with the viral genome (CPV_Genome) and forms genome bound NS1 (Bound NS1). Similar reaction takes place between bleached, non-fluorescent NS1 (Bleached NS1) and the viral genome. This reaction forms non-fluorescent bound NS1 (Bleached Bound NS1). Bleaching laser induces the bleaching reaction, where fluorescent NS1 forms non-fluorescent bleached NS1 or where bound NS1 forms bleached, bound NS1. Imaging laser reacts with fluorescent forms of NS1 and simulates the bleaching reaction caused by the confocal imaging.
(0.24 MB TIF)

Figure S5 Schematic presentation of PAGFP-VP2 Virtual Cell model. Schematic representation of the molecular species (green) and reactions between them (yellow) in PAGFP-VP2 activation study simulations. Activation laser reacts with dark, mobile and immobile capsids and leads to formation of bright capsids. Imaging laser simulates the bleaching caused by the confocal imaging.
(0.19 MB TIF)

Figure S6 Intracellular distribution of EYFP and histone H2B in infected and non-infected cells. Confocal images show the distribution of EYFP and H2B-EYFP in (A) in infected and (B) non-infected cells at 24 h p.i. Line profile analysis revealed intensity profiles through the nuclear region. Scale bar, 5 µm.
(1.18 MB TIF)

Figure S7 Free EYFP and EGFP diffusion in nuclei of living cells. Diffusion time of free EYFP was measured with FFM in the nucleoplasm of living NLFK cells at various positions. (A) Representative NLFK cell showing 3 measurement points and (B) the measured autocorrelation curves, respectively. (C) Summary of measured EYFP diffusion coefficients in NLKF cell nuclei and EGFP diffusion coefficients in HEK293, HeLa, T98G and TP366 nuclei. Scale bar, 5 µm.
(3.87 MB TIF)

Text S1 Supplementary Material and Methods
(0.12 MB DOC)

## References

[1] Mettenleiter TC (2002). Herpesvirus assembly and egress.. J Virol.

[2] Berk AJ (2005). Recent lessons in gene expression, cell cycle control, and cell biology from adenovirus.. Oncogene.

[3] Taylor TJ, Knipe DM (2004). Proteomics of herpes simplex virus replication compartments: Association of cellular DNA replication, repair, recombination, and chromatin remodeling proteins with ICP8.. J Virol.

[4] Everett RD (2001). DNA viruses and viral proteins that interact with PML nuclear bodies.. Oncogene.

[5] Weitzman MD, Carson CT, Schwartz RA, Lilley CE (2004). Interactions of viruses with the cellular DNA repair machinery.. DNA Repair (Amst).

[6] Lamond AI, Spector DL (2003). Nuclear speckles: A model for nuclear organelles.. Nat Rev Mol Cell Biol.

[7] Cremer T, Cremer M, Dietzel S, Muller S, Solovei I (2006). Chromosome territories—a functional nuclear landscape.. Curr Opin Cell Biol.

[8] Lanctot C, Cheutin T, Cremer M, Cavalli G, Cremer T (2007). Dynamic genome architecture in the nuclear space: Regulation of gene expression in three dimensions.. Nat Rev Genet.

[9] Cremer C, Munkel C, Granzow M, Jauch A, Dietzel S (1996). Nuclear architecture and the induction of chromosomal aberrations.. Mutat Res.

[10] Cremer T, Kreth G, Koester H, Fink RH, Heintzmann R (2000). Chromosome territories, interchromatin domain compartment, and nuclear matrix: An integrated view of the functional nuclear architecture.. Crit Rev Eukaryot Gene Expr.

[11] Albiez H, Cremer M, Tiberi C, Vecchio L, Schermelleh L (2006). Chromatin domains and the interchromatin compartment form structurally defined and functionally interacting nuclear networks.. Chromosome Res.

[12] Carter DR, Eskiw C, Cook PR (2008). Transcription factories.. Biochem Soc Trans.

[13] Handwerger KE, Gall JG (2006). Subnuclear organelles: New insights into form and function.. Trends Cell Biol.

[14] Misteli T (2007). Beyond the sequence: Cellular organization of genome function.. Cell.

[15] Tseng Y, Lee JS, Kole TP, Jiang I, Wirtz D (2004). Micro-organization and visco-elasticity of the interphase nucleus revealed by particle nanotracking.. J Cell Sci.

[16] Guigas G, Kalla C, Weiss M (2007). Probing the nanoscale viscoelasticity of intracellular fluids in living cells.. Biophys J.

[17] Zeskind BJ, Jordan CD, Timp W, Trapani L, Waller G (2007). Nucleic acid and protein mass mapping by live-cell deep-ultraviolet microscopy.. Nat Methods.

[18] Gorisch SM, Wachsmuth M, Toth KF, Lichter P, Rippe K (2005). Histone acetylation increases chromatin accessibility.. J Cell Sci.

[19] Gorisch SM, Wachsmuth M, Ittrich C, Bacher CP, Rippe K (2004). Nuclear body movement is determined by chromatin accessibility and dynamics.. Proc Natl Acad Sci U S A.

[20] Tsao J, Chapman MS, Agbandje M, Keller W, Smith K (1991). The three-dimensional structure of canine parvovirus and its functional implications.. Science.

[21] Reed AP, Jones EV, Miller TJ (1988). Nucleotide sequence and genome organization of canine parvovirus.. J Virol.

[22] Parrish CR (1991). Mapping specific functions in the capsid structure of canine parvovirus and feline panleukopenia virus using infectious plasmid clones.. Virology.

[23] Jindal HK, Yong CB, Wilson GM, Tam P, Astell CR (1994). Mutations in the NTP-binding motif of minute virus of mice (MVM) NS-1 protein uncouple ATPase and DNA helicase functions.. J Biol Chem.

[24] Christensen J, Cotmore SF, Tattersall P (1995). Minute virus of mice transcriptional activator protein NS1 binds directly to the transactivation region of the viral P38 promoter in a strictly ATP-dependent manner.. J Virol.

[25] Christensen J, Cotmore SF, Tattersall P (2001). Minute virus of mice initiator protein NS1 and a host KDWK family transcription factor must form a precise ternary complex with origin DNA for nicking to occur.. J Virol.

[26] Cotmore SF, Christensen J, Nuesch JP, Tattersall P (1995). The NS1 polypeptide of the murine parvovirus minute virus of mice binds to DNA sequences containing the motif [ACCA]2-3.. J Virol.

[27] Nuesch JP, Cotmore SF, Tattersall P (1995). Sequence motifs in the replicator protein of parvovirus MVM essential for nicking and covalent attachment to the viral origin: Identification of the linking tyrosine.. Virology.

[28] Cziepluch C, Lampel S, Grewenig A, Grund C, Lichter P (2000). H-1 parvovirus-associated replication bodies: A distinct virus-induced nuclear structure.. J Virol.

[29] Iwahori S, Ikeda M, Kobayashi M (2004). Association of Sf9 cell proliferating cell nuclear antigen with the DNA replication site of autographa californica multicapsid nucleopolyhedrovirus.. J Gen Virol.

[30] Daikoku T, Kudoh A, Sugaya Y, Iwahori S, Shirata N (2006). Postreplicative mismatch repair factors are recruited to epstein-barr virus replication compartments.. J Biol Chem.

[31] Christensen J, Cotmore SF, Tattersall P (1997). A novel cellular site-specific DNA-binding protein cooperates with the viral NS1 polypeptide to initiate parvovirus DNA replication.. J Virol.

[32] Nash K, Chen W, McDonald WF, Zhou X, Muzyczka N (2007). Purification of host cell enzymes involved in adeno-associated virus DNA replication.. J Virol.

[33] Dross N, Spriet C, Zwerger M, Muller G, Waldeck W (2009). Mapping eGFP oligomer mobility in living cell nuclei.. PLoS ONE.

[34] Suikkanen S, Saajarvi K, Hirsimaki J, Valilehto O, Reunanen H (2002). Role of recycling endosomes and lysosomes in dynein-dependent entry of canine parvovirus.. J Virol.

[35] Ihalainen TO, Niskanen EA, Jylhava J, Turpeinen T, Rinne J (2007). Dynamics and interactions of parvoviral NS1 protein in the nucleus.. Cell Microbiol.

[36] Patterson GH, Lippincott-Schwartz J (2002). A photoactivatable GFP for selective photolabeling of proteins and cells.. Science.

[37] Parker JS, Parrish CR (1997). Canine parvovirus host range is determined by the specific conformation of an additional region of the capsid.. J Virol.

[38] Hauck B, Zhao W, High K, Xiao W (2004). Intracellular viral processing, not single-stranded DNA accumulation, is crucial for recombinant adeno-associated virus transduction.. J Virol.

[39] Parker JS, Murphy WJ, Wang D, O'Brien SJ, Parrish CR (2001). Canine and feline parvoviruses can use human or feline transferrin receptors to bind, enter, and infect cells.. J Virol.

[40] Essers J, Theil AF, Baldeyron C, van Cappellen WA, Houtsmuller AB (2005). Nuclear dynamics of PCNA in DNA replication and repair.. Mol Cell Biol.

[41] Braga J, Desterro JM, Carmo-Fonseca M (2004). Intracellular macromolecular mobility measured by fluorescence recovery after photobleaching with confocal laser scanning microscopes.. Mol Biol Cell.

[42] Sprague BL, Pego RL, Stavreva DA, McNally JG (2004). Analysis of binding reactions by fluorescence recovery after photobleaching.. Biophys J.

[43] Christensen J, Tattersall P (2002). Parvovirus initiator protein NS1 and RPA coordinate replication fork progression in a reconstituted DNA replication system.. J Virol.

[44] Young PJ, Newman A, Jensen KT, Burger LR, Pintel DJ (2005). Minute virus of mice small non-structural protein NS2 localizes within, but is not required for the formation of, smn-associated autonomous parvovirus-associated replication bodies.. J Gen Virol.

[45] de Oliveira AP, Glauser DL, Laimbacher AS, Strasser R, Schraner EM (2008). Live visualization of herpes simplex virus type 1 compartment dynamics.. J Virol.

[46] Blissard GW (1996). Baculovirus—insect cell interactions.. Cytotechnology.

[47] Verschure PJ, van der Kraan I, Manders EM, Hoogstraten D, Houtsmuller AB (2003). Condensed chromatin domains in the mammalian nucleus are accessible to large macromolecules.. EMBO Rep.

[48] Gorisch SM, Richter K, Scheuermann MO, Herrmann H, Lichter P (2003). Diffusion-limited compartmentalization of mammalian cell nuclei assessed by microinjected macromolecules.. Exp Cell Res.

[49] Gilbert L, Toivola J, Valilehto O, Saloniemi T, Cunningham C (2006). Truncated forms of viral VP2 proteins fused to EGFP assemble into fluorescent parvovirus-like particles.. J Nanobiotechnology.

[50] Riolobos L, Reguera J, Mateu MG, Almendral JM (2006). Nuclear transport of trimeric assembly intermediates exerts a morphogenetic control on the icosahedral parvovirus capsid.. J Mol Biol.

[51] Gilbert L, Toivola J, Lehtomaki E, Donaldson L, Kapyla P (2004). Assembly of fluorescent chimeric virus-like particles of canine parvovirus in insect cells.. Biochem Biophys Res Commun.

[52] Seksek O, Biwersi J, Verkman AS (1997). Translational diffusion of macromolecule-sized solutes in cytoplasm and nucleus.. J Cell Biol.

[53] Seisenberger G, Ried MU, Endress T, Buning H, Hallek M (2001). Real-time single-molecule imaging of the infection pathway of an adeno-associated virus.. Science.

[54] Muller DE, Siegl G (1983). Maturation of parvovirus LuIII in a subcellular system. II. isolation and characterization of nucleoprotein intermediates.. J Gen Virol.

[55] Janicki SM, Spector DL (2003). Nuclear choreography: Interpretations from living cells.. Curr Opin Cell Biol.

[56] Monier K, Armas JC, Etteldorf S, Ghazal P, Sullivan KF (2000). Annexation of the interchromosomal space during viral infection.. Nat Cell Biol.

[57] Nagamine T, Kawasaki Y, Abe A, Matsumoto S (2008). Nuclear marginalization of host cell chromatin associated with expansion of two discrete virus-induced subnuclear compartments during baculovirus infection.. J Virol.

[58] Oh J, Fraser NW (2008). Temporal association of the herpes simplex virus genome with histone proteins during a lytic infection.. J Virol.

[59] Wachsmuth M, Waldeck W, Langowski J (2000). Anomalous diffusion of fluorescent probes inside living cell nuclei investigated by spatially-resolved fluorescence correlation spectroscopy.. J Mol Biol.

[60] Kues T, Peters R, Kubitscheck U (2001). Visualization and tracking of single protein molecules in the cell nucleus.. Biophys J.

[61] Richter K, Nessling M, Lichter P (2008). Macromolecular crowding and its potential impact on nuclear function.. Biochim Biophys Acta.

[62] Wedemeier A, Merlitz H, Wu CX, Langowski J (2007). Modeling diffusional transport in the interphase cell nucleus.. J Chem Phys.

[63] Saxton MJ (1989). Lateral diffusion in an archipelago. distance dependence of the diffusion coefficient.. Biophys J.

[64] von Hippel PH, Berg OG (1989). Facilitated target location in biological systems.. J Biol Chem.

[65] Bashir T, Rommelaere J, Cziepluch C (2001). In vivo accumulation of cyclin A and cellular replication factors in autonomous parvovirus minute virus of mice-associated replication bodies.. J Virol.

[66] Marenduzzo D, Finan K, Cook PR (2006). The depletion attraction: An underappreciated force driving cellular organization.. J Cell Biol.

[67] Braga J, McNally JG, Carmo-Fonseca M (2007). A reaction-diffusion model to study RNA motion by quantitative fluorescence recovery after photobleaching.. Biophys J.

[68] Lukacs GL, Haggie P, Seksek O, Lechardeur D, Freedman N (2000). Size-dependent DNA mobility in cytoplasm and nucleus.. J Biol Chem.

[69] Cotmore SF, Gottlieb RL, Tattersall P (2007). Replication initiator protein NS1 of the parvovirus minute virus of mice binds to modular divergent sites distributed throughout duplex viral DNA.. J Virol.

[70] Naryzhny SN, Zhao H, Lee H (2005). Proliferating cell nuclear antigen (PCNA) may function as a double homotrimer complex in the mammalian cell.. J Biol Chem.

[71] Solomon DA, Cardoso MC, Knudsen ES (2004). Dynamic targeting of the replication machinery to sites of DNA damage.. J Cell Biol.

[72] Phair RD, Scaffidi P, Elbi C, Vecerova J, Dey A (2004). Global nature of dynamic protein-chromatin interactions in vivo: Three-dimensional genome scanning and dynamic interaction networks of chromatin proteins.. Mol Cell Biol.

[73] Cotmore SF, Tattersall P (2005). Genome packaging sense is controlled by the efficiency of the nick site in the right-end replication origin of parvoviruses minute virus of mice and LuIII.. J Virol.

[74] Jackson DA, Pombo A (1998). Replicon clusters are stable units of chromosome structure: Evidence that nuclear organization contributes to the efficient activation and propagation of S phase in human cells.. J Cell Biol.

[75] Norio P, Schildkraut CL (2004). Plasticity of DNA replication initiation in epstein-barr virus episomes.. PLoS Biol.

[76] Yamashita T, Arens M, Green M (1975). Adenovirus deoxyribonucleic acid replication. II. synthesis of viral deoxyribonucleic acid in vitro by a nuclear membrane fraction from infected KB cells.. J Biol Chem.

