# Supplementary figures and images for: Parvovirus Induced Alterations in Nuclear Architecture and Dynamics
Source: PLoS One. 2009 Jun 17;4(6):e5948. doi: 10.1371/journal.pone.0005948 (PMC2694274; doi:10.1371/journal.pone.0005948)

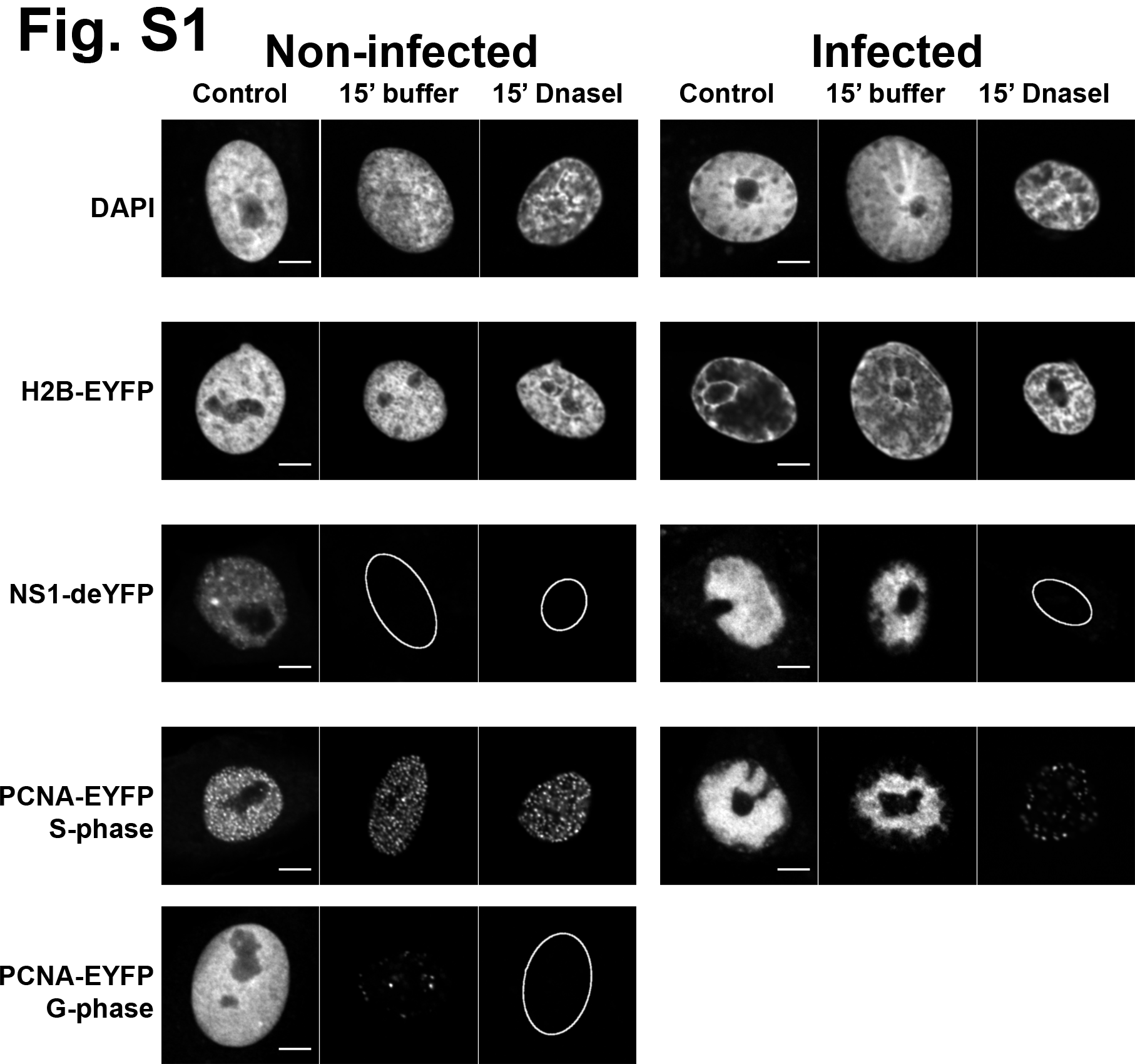

Supplement: Figure S1 — Effect of DNaseI treatment on non-infected and infected NLFK cells. Fixed cell confocal microscopy images of dsDNA (DAPI label), chromatin (H2B-EYFP), NS1-deYFP and PCNA-EYFP after permeabilization and treatment with buffer or DNaseI. Unapparent nuclei are encircled. Scale bars, 5 µm. (0.53 MB TIF) [file pone.0005948.s002.tif]

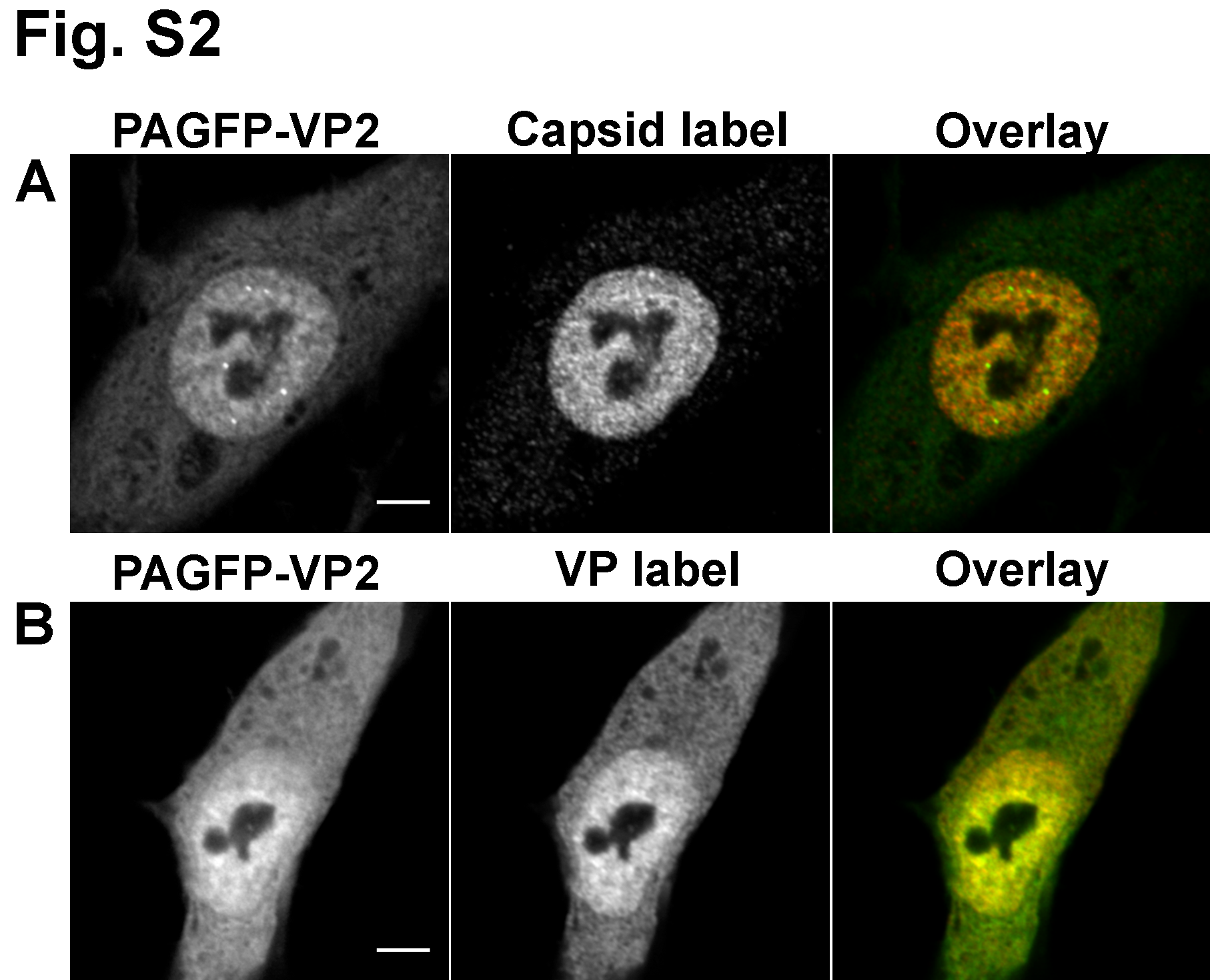

Supplement: Figure S2 — Intracellular localization of capsids and viral VP proteins in PAGFP-VP2 expressing cells. NLFK cells expressing PAGFP-VP2 were immunolabelled with antibodies that recognize intact capsids or VP proteins. Distribution of PAGFP-VP2 (green) together with (A) capsid MAb (red) or (B) VP Ab (red). Secondary antibodies used were Alexa-555-labled anti-mouse IgG and anti-rabbit IgG. Scale bars, 5 µm. (1.10 MB TIF) [file pone.0005948.s003.tif]

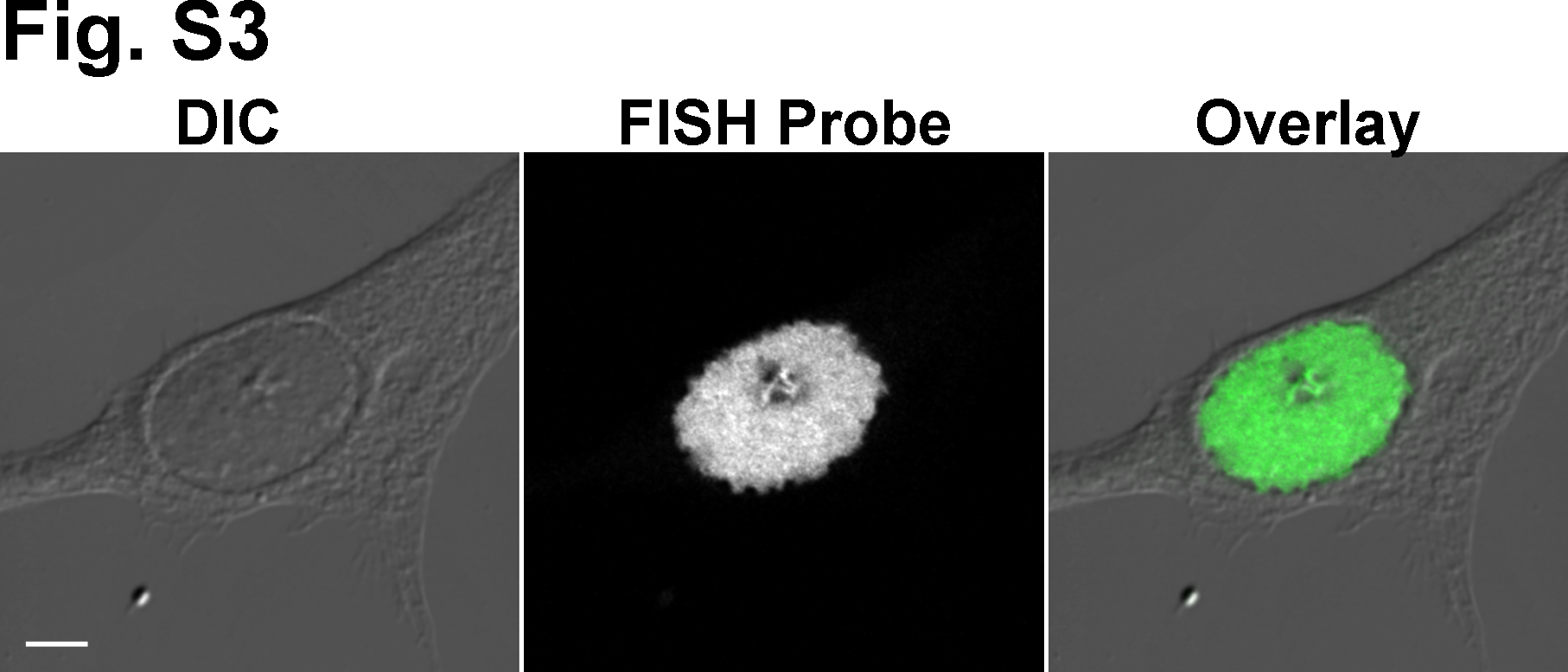

Supplement: Figure S3 — Fluorescence in situ labelling of viral genome. Infected cells were labelled with CPV genome specific FISH probe at 24 h p.i. The FISH probe labelled the viral replication compartment inside the nucleus. Scale bar, 5 µm. (0.54 MB TIF) [file pone.0005948.s004.tif]

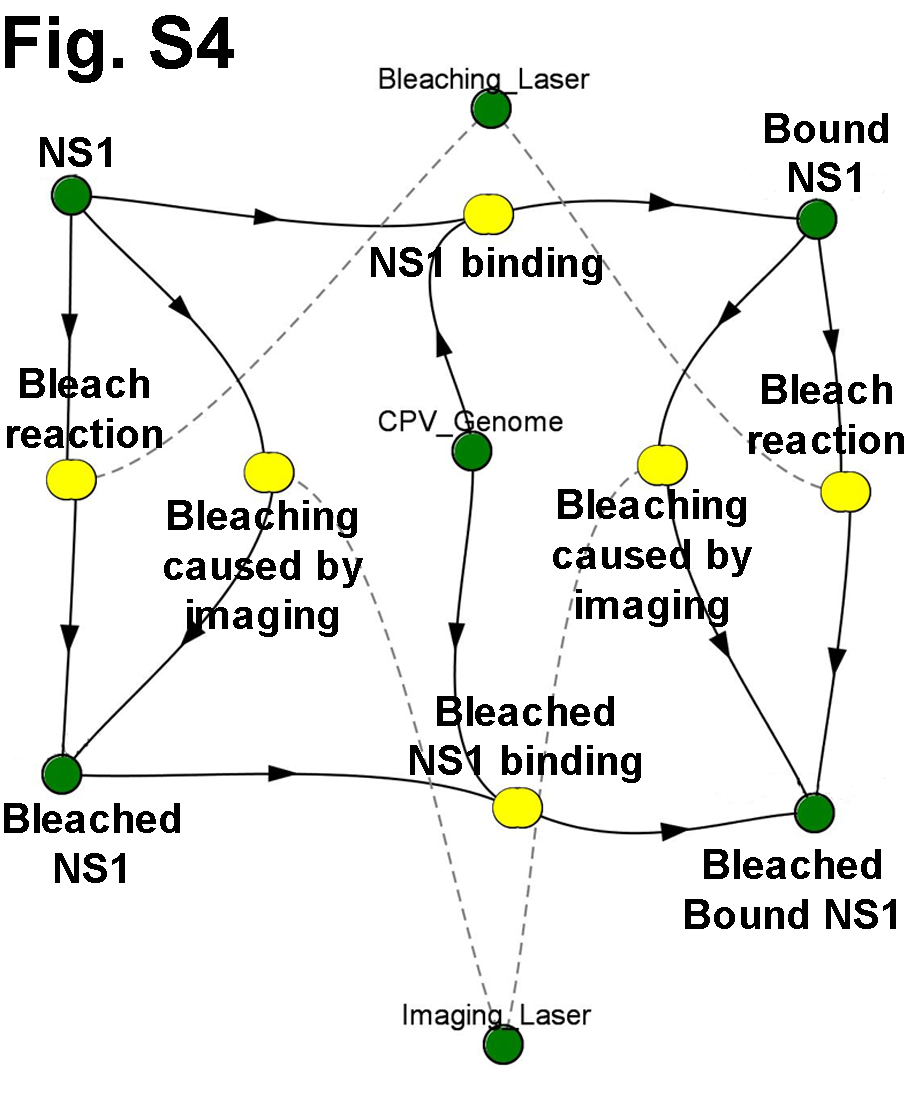

Supplement: Figure S4 — Schematic presentation of NS1-deYFP Virtual Cell model. Schematic representation of the Virtual Cell simulation showing the molecular species (green) and reactions between them (yellow). In the model, fluorescent NS1-deYFP (NS1) reacts with the viral genome (CPV_Genome) and forms genome bound NS1 (Bound NS1). Similar reaction takes place between bleached, non-fluorescent NS1 (Bleached NS1) and the viral genome. This reaction forms non-fluorescent bound NS1 (Bleached Bound NS1). Bleaching laser induces the bleaching reaction, where fluorescent NS1 forms non-fluorescent bleached NS1 or where bound NS1 forms bleached, bound NS1. Imaging laser reacts with fluorescent forms of NS1 and simulates the bleaching reaction caused by the confocal imaging. (0.24 MB TIF) [file pone.0005948.s005.tif]

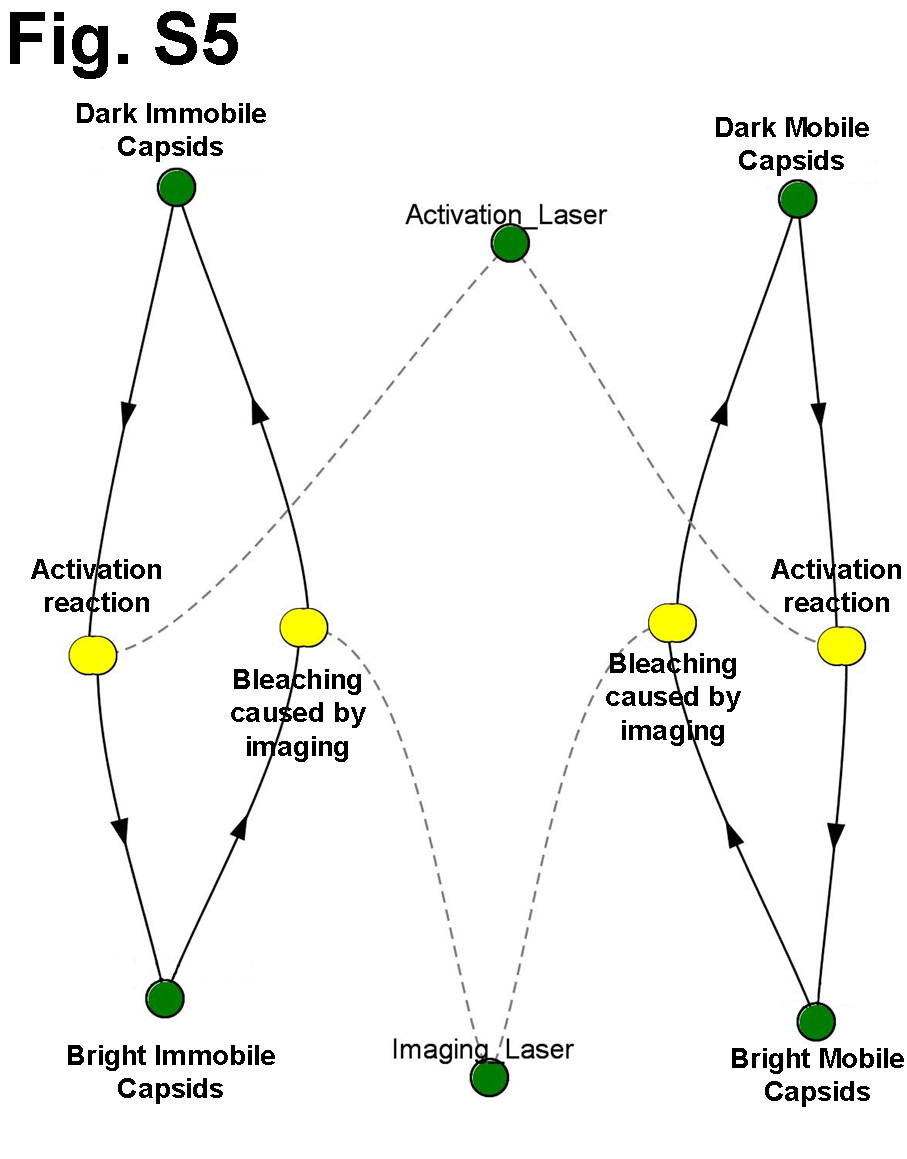

Supplement: Figure S5 — Schematic presentation of PAGFP-VP2 Virtual Cell model. Schematic representation of the molecular species (green) and reactions between them (yellow) in PAGFP-VP2 activation study simulations. Activation laser reacts with dark, mobile and immobile capsids and leads to formation of bright capsids. Imaging laser simulates the bleaching caused by the confocal imaging. (0.19 MB TIF) [file pone.0005948.s006.tif]

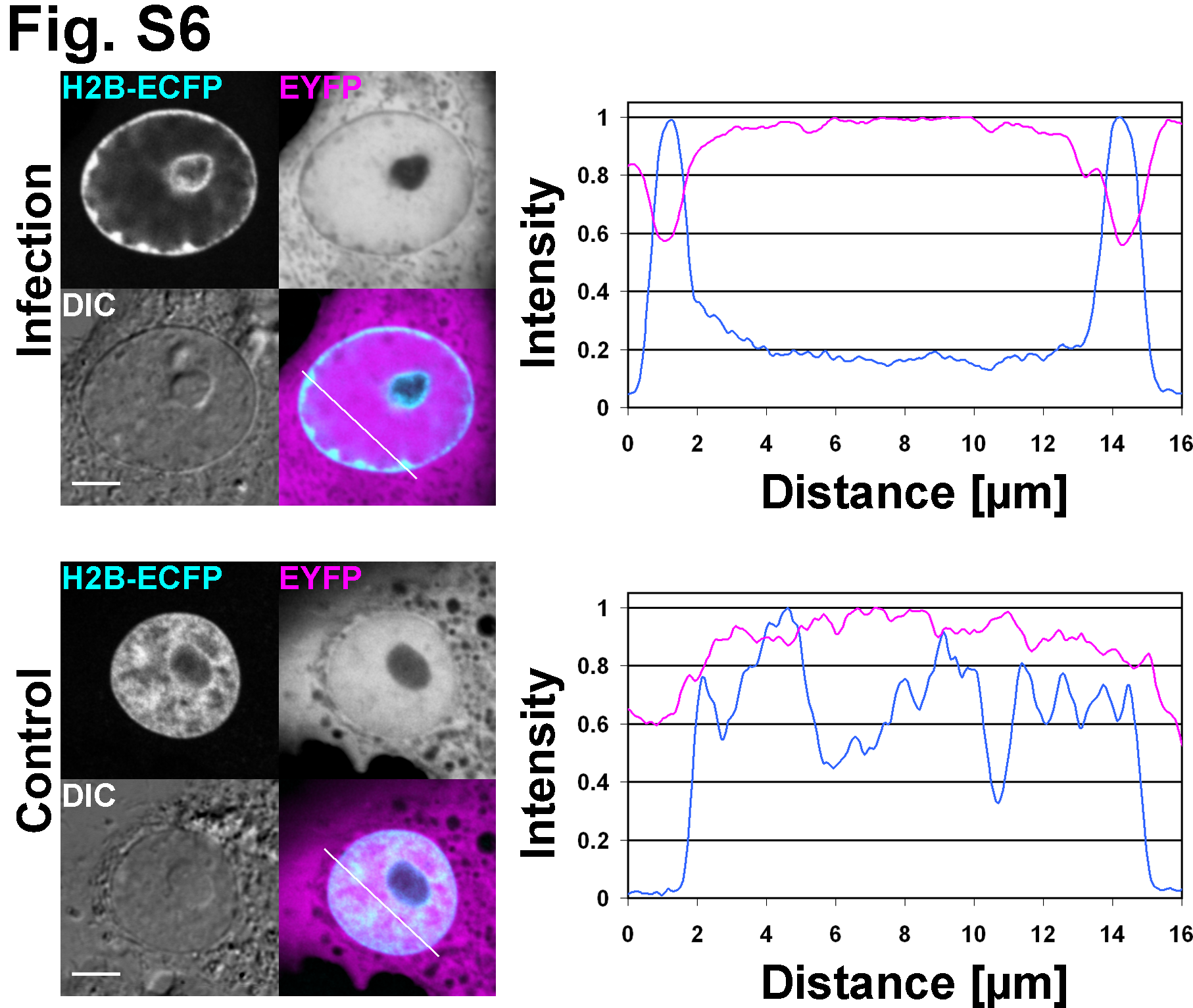

Supplement: Figure S6 — Intracellular distribution of EYFP and histone H2B in infected and non-infected cells. Confocal images show the distribution of EYFP and H2B-EYFP in (A) in infected and (B) non-infected cells at 24 h p.i. Line profile analysis revealed intensity profiles through the nuclear region. Scale bar, 5 µm. (1.18 MB TIF) [file pone.0005948.s007.tif]

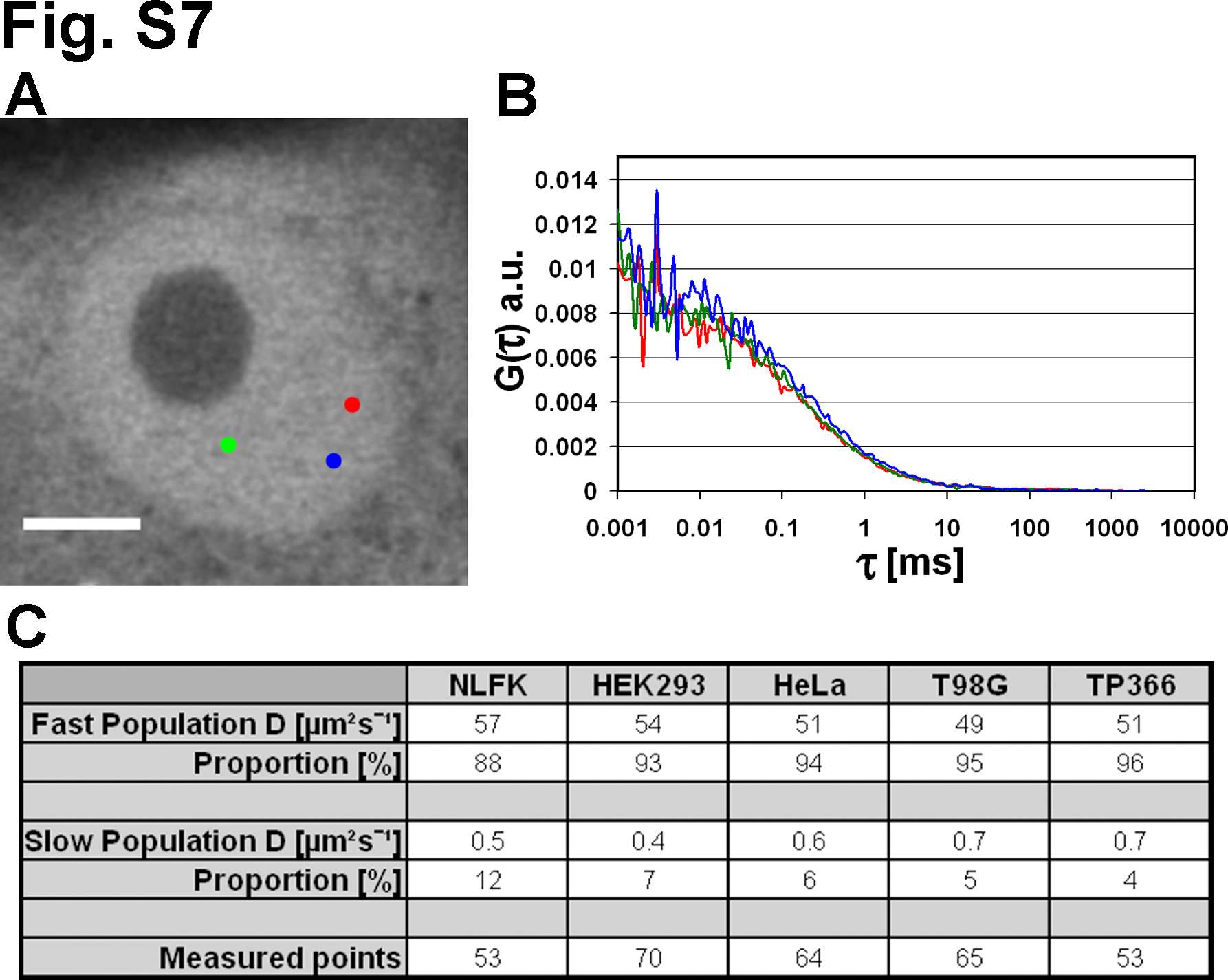

Supplement: Figure S7 — Free EYFP and EGFP diffusion in nuclei of living cells. Diffusion time of free EYFP was measured with FFM in the nucleoplasm of living NLFK cells at various positions. (A) Representative NLFK cell showing 3 measurement points and (B) the measured autocorrelation curves, respectively. (C) Summary of measured EYFP diffusion coefficients in NLKF cell nuclei and EGFP diffusion coefficients in HEK293, HeLa, T98G and TP366 nuclei. Scale bar, 5 µm. (3.87 MB TIF) [file pone.0005948.s008.tif]
