# Supplementary material for: Parvovirus Induced Alterations in Nuclear Architecture and Dynamics
Source: PLoS One. 2009 Jun 17;4(6):e5948. doi: 10.1371/journal.pone.0005948 (PMC2694274; doi:10.1371/journal.pone.0005948)
Supplement: Text S1 — Supplementary Material and Methods (0.12 MB DOC) [file pone.0005948.s009.doc]

**Supporting Text S1**

**Supplementary Material and Methods**

**DNaseI Digestion Assays**

Studies were performed with wild type NLFK cells, stably H2B-EYFP or PCNA-EYFP expressing cells, or transiently NS1-deYFP expressing cells. The cells were washed with 20 mM Hepes buffer (110 mM potassium acetate, 5 mM sodium acetate and 2 mM magnesium acetate, pH 7.3) and permeabilized with the washing buffer containing 0.5 % Triton-X100. Then, the cells were then treated either with the washing buffer or buffer containing 130 ng/ml of DNaseI for 15 minutes in 37 °C. Permeabilization, washing and DNaseI treatments were done in the presence of protease inhibitors. After fixation with 4 % PFA for 15 minutes at RT the cells were embedded in Mowiol containing Dabco antifade reagent (Sigma) or in Prolong Gold containing DAPI (Molecular Probes).

**Detection of CPV genome by Fluorescence In Situ Hybridization**

First, primers were designed to amplify 1000 bp area from CPV infectious clone.The primers used were 5’-TGGATGTGAAGAAAGACCTG-3’ and 5’-GGTTGAACTGCTCCATCACTC-3’. The PCR reaction contained aminoallyl-dUTP nucleotides (Fermentas international Inc, Burlington, Canada). The PCR product was isolated from agarose gel and labelled with ATTO-488-NHS ester according to the manufacturer's instructions (Sigma). Infected cells were grown on cover slips and fixed for 10 min with methanol:acetic acid (3:1) at 16 h p.i., dehydrated in increasing ethanol series (70, 80 and 100 %) for 1 min each and then air dried for 5 min. Samples and hybridization probe was denatured at 72 °C. Hybridization was performed at 37 °C for 15 h in 2x SSC containing 50 % formamide and 30 % dextran sulphate. After hybridization, samples were washed with 2xSSC-formamide and with 2xSSC, embedded in Mowiol and imaged on a confocal microscope.

**FRAP experiments**

The FRAP experiments were performed on a laser scanning confocal microscope LSM510, Axiovert 100M (Zeiss, Jena, Germany) using Zeiss Plan-Neofluar 63x (NA=1.25) oil immersion objective. The objective and the sample stage were heated to 37 °C. The image size was adjusted to 256 x 256 pixels, with a pixel size of 95 nm (zoom factor 6). The pinhole size was adjusted between 1-2 Airy units. The 514 nm laser line was used for EYFP excitation and the emitted fluorescence was detected using a 530-600 nm bandpass filter. In the bleach process the laser intensity was set to 100 % by using an acousto optical tunable filter and imaging was performed with laser intensities between 0.1-2 %. The bleaching iterations were adjusted to achieve optimal bleaching within a minimum time. The bleaching region of interest, (ROI) was placed on the middle of the image. With a circular ROI, the size was measured by bleaching fixed cells and measuring the full width at half maximum, which was used as the ROI diameter. When rectangular area was bleached, the ROI width was measured from the first image after the bleach. Table 1 summarizes the bleaching and imaging parameters of the various constructs.

| **FRAP experiment** | **Bleach ROI Size** | **Iterations** | **Pinhole Size [Airy]** | **Imaging Laser Power [%]** | **Frame Interval** | **Imaging area** |
| --- | --- | --- | --- | --- | --- | --- |
| **EYFP** | 0.88±0.12 µm  **○** | 7 | 2 | 0.1 | 25 ms | 1.9 x 24.4 µm **□** |
| **NS1-deYFP** | 1.07± 0.18 µm  **○** | 7 | 2 | 0.1 | 500 ms | Full Frame |
| **PCNA-EYFP  non-infected** | 0.86±0.15 µm  **○** | 8 | 1.5 | 2 | 65 ms | 3.8 x 24.4 µm **□** |
| **PCNA-EYFP infected** | 0.86±0.15 µm  **○** | 7 | 1.5 | 1 | 250 ms | Full Frame |
| **H2B-EYFP** | 1.28 µm x 24.4 µm  **□** | 10 | 1 | 0.1 | 1 s | Full Frame |

Table 1. Parameters for the FRAP experiments.

**Photoactivable GFP-VP2.**

The activation studies were performed with a FV1000 laser scanning confocal microscope on an IX-81 inverted microscope (Olympus, Japan) using an Olympus UPLSAPO 60x (NA=1.2) water immersion objective. The confocal microscope was equipped with two point scanners. The 405 nm activation laser line is attached to the second scanner while the 488 nm laser line used in the imaging was guided to the main imaging scanner. The correction collar of the objective was adjusted to the coverslip thickness for every cell culture dish and the sample stage was heated to 37 °C prior to the experiments. The image size was 256x256 pixels, with a pixel size adjusted to 100 nm and a pinhole of 1 Airy unit. A circular ROI of with a diameter of 2 µm was used in the activation process and the 405 nm laser line illuminated the ROI for a total time of 3 s. Images were captured simultaneously by the main imaging scanner using a excitation wavelength of 488 nm excitation and the emission was detected with a 500-600 nm bandpass-filter. The interframe time was set to510 ms and the fluorescence redistribution was followed for a total of 63 s.

**FRAP data normalization, fitting and Virtual Cell modelling**

When the recovery was followed by imaging the whole nucleus, the data were normalized using equation 1 [1]

$I\left(t\right)=\frac{\frac{ROI\left(t\right)}{ROI\left(0\right)}}{\frac{Nucleus\left(t\right)}{Nucleus\left(0\right)}}=\frac{Nucleus\left(0\right)}{ROI\left(0\right)}*\frac{ROI\left(t\right)}{Nucleus\left(t\right)}$ (Eq. 1)

where *ROI(0)* is the average intensity of the bleach region before the bleach, *ROI(t)* the average intensity of the bleach region at a time point t, *Nucleus(0)* the average nuclear fuorescence before the bleach and *Nucleus(t)* the average nuclear fluorescence at time point t.

When smaller imaging area was used to achieve a higher frame rate, this normalization method was no longer possible. In these experiments the fluorescence loss caused by the bleach pulse was measured afterwards by imaging the whole nucleus and bleaching similar area with identical bleaching parameters. From the images taken before and after the bleach, the amount of bleaching (marked as B) in equation 2 can be calculated. The loss of fluorescence while following the recovery was measured by performing a similar FRAP experiments without the bleach pulse. These data showed how much of the fluorescence was lost due to the imaging process (noted Im(t) in equation 2). Both of these were combined with equation 1 to result in a second normalization method:

$I\left(t\right)\left\{\begin{matrix} \frac{\mathrm{Im}(o)}{ROI(0)}*\frac{ROI(t)}{\mathrm{Im}(t)},t<bleachpulse \\ \\ \frac{\mathrm{Im}(o)*B}{ROI(0)}*\frac{ROI(t)}{\mathrm{Im}(t)},t>bleachpulse \end{matrix}\right.$ (Eq. 2)

where *Im(0)* is the average intensity of the imaging region before the bleach, *Im(t)* the average intensity of the imaging region at a time point t, measured separately from the FRAP experiment, *ROI(0)* the average intensity of the bleach region before the bleach, *ROI(t)* the average intensity of the bleach region at a time point t in the FRAP experiment and B the total amount of bleaching inside the nucleus, caused by the bleach pulse, measured separately from the FRAP experiment. The FRAP data were normalized and averaged, usually using 8 to 15 cells for the averaging process.

**FRAP data fitting**

The data from quantitative EYFP, NS1-deYFP and PCNA-EYFP FRAP experiments were fitted to a recovery model as described by Sprague et al. [2]. The free diffusion constant of PCNA-EYFP and NS1-deYFP were estimated by using the relation$D_{f}\alpha M^{\frac{-1}{3}}$, where *M* is the mass of the protein. According to this relation, we can write

(Eq. 3)

where $D_{f}\left(P+T\right)$ is the diffusion constant of the fusion protein, $D_{f}\left(T\right)$ the measured free diffusion constant of the fluorescent tag, $M\left(P+T\right)$ the mass of the fusion protein and $M\left(T\right)$ the mass of the free fluorescent tag. For free diffusion, the data were fitted by the expression

$I(t)=e^{-\frac{\tau_{D}}{2t}}\left[I_{0}\left(\frac{\tau_{D}}{2t}\right)+I_{1}\left(\frac{\tau_{D}}{2t}\right)\right]$ (Eq. 4)

with $\tau_{D}=w^{2}/D_{f}$. In equation 4, $I(t)$ is the normalized fluorescence, *t* the time, $I_{0}$ and $I_{1}$ modified Bessel functions, *w* the bleach spot radius, and $D_{f}$ the diffusion constant. The binding reactions were assumed to be in the form of

The ligand binds to the substrate with an on-rate k_on_ and forms a complex, which then dissociates with a reaction off-rate k_off_. This process can be described as $\frac{\left[Substrate\right]*k_{on}}{k_{off}}=\frac{\left[Ligand\right]}{\left[Complex\right]}\to\frac{k_{on}^{*}}{k_{off}}=\frac{\left[Ligand\right]}{\left[Complex\right]}$ , where  *k^*^_on_* is the pseudo on-rate of the reaction. In the case of binding reactions in FRAP recovery, an inverse Laplace transformation of the expression

$I(t)=\frac{1}{p}-\frac{F_{eq}}{p}\left(1-2K_{1}(qw)I_{1}(qw)\right)*\left(1+\frac{k_{on}^{*}}{p+k_{off}}\right)-\frac{C_{eq}}{p+k_{off}}$ (Eq. 5)

with $q=\sqrt{\left(\frac{p}{D_{f}}\right)\left(1+\frac{k_{on}^{*}}{p+k_{off}}\right)}$, was used. In equation 5, $I_{1}$and $K_{1}$are modified Bessel functions and *p* is a variable which becomes time when the inverse transform of equation 2 is performed to compute the FRAP recovery. *F_eq_ = k_off_ / (k^*^_on_ + k_off_)* and *C_eq_ = k^*^_on_ / (k^*^_on_ + k_off_)* correspond respectively to the free and bound equilibrium fractions of the fusion proteins, and $k_{off}$is the binding off-rate and  *k^*^_on_* the pseudo on-rate of the binding. The fitting procedure was performed as described previously in [3].

**Virtual Cell Modeling of the FRAP recovery data**

FRAP recoveries were also modelled with the Virtual Cell software [4]. The nucleus was assumed to have an ellipsoidal shape, with a width of 10 µm and a height of 15 µm. The time step of the simulation was set to 1 ms and images were collected with the same frame rate as in the FRAP studies. Bleaching reaction was modelled by a laser light induced mass action reaction. The bleaching ROI was approximated to correspond in geometry and size to the one in the FRAP experiments. The length of the bleach pulse was adjusted to 60 ms, as in the FRAP experiments. In addition, bleaching due to imaging laser was modelled by a second continuous bleaching reaction in the nucleus. The frame time on the confocal microscope is about 200 ms, and with the bleaching ROI being in the middle of the image, it was assumed that the bleach region was imaged about 100 ms after the bleach. This value was also used in the simulations as the first recovery image timepoint. The binding reactions were modelled as a mass action reaction, in which the free ligand reacted with the substrate and formed the complex. The amount of free binding sites was set at 1 µM, in this situation, *k^*^_on_ =*[*sites*]*⋅k_on_ = k_on_*.

The free diffusion constant was D = 18 µm^2^/s for NS1-deYFP. In NS1-deYFP modelling the mobile binding sites had a diffusion constant of D = 0.01 µm^2^/s. The binding reaction k_on_ and k_off_ rates were changed in the modelling process until the model fitted with the measured data. The reaction map is shown in supplementary Figure S4.

**Fluorescence Fluctuation Microscopy (FFM)**

We used a setup built in the laboratory of Dr. J. Langowski, the Fluorescence Fluctuation Microscope (FFM) [5], which is a combination of Fluorescence Correlation Spectroscopy (FCS) and Confocal Laser Scanning Microscopy (CLSM). It combines a FCS module with a galvanometer scanning unit, attached to the side port of an inverted IX-70 microscope (Olympus, Hamburg, Germany) equipped with an UplanApo / IR 60x water immersion objective ,with a numerical aperture (NA) of 1.2 [5, 6].

For fluorescence excitation, we used an argon-krypton laser from CVI Melles Griot (Bensheim, Germany), using the 488 nm laser line for the excitation of YFP. The emitted fluorescence from YFP was detected from 515 to 545 nm with an avalanche photodiode (APD) (SPCM-AQR-13, Perkin-Elmer, Wellesley, USA), after passing appropriate dichroic mirrors and filters for spectral separation and selection. FCS measurements were carried out at laser intensities from 5 to 9 kW⋅cm^-2^ and laser-power was adjusted using a polychromatic acousto optical modulator AOTF Nc (AA Opto Electronic, France), which allows precise control over the laser power.

The signals coming from the APDs were fed into an ALV-5000/E correlator card (ALV Laser GmbH, Langen, Germany), which records the intensity fluctuations and calculate their associated autocorrelation function simultaneously and almost in real-time.

The system was carefully calibrated as described in [7] to allow precise and reproducible FCS-measurements.

**FFM data acquisition and sorting**

Intracellular measurements were all performed at 37 °C, in a 5 % CO_2_ humidified atmosphere, in a incubator chamber (EMBL, Heidelberg, Germany) surrounding the whole microscope, with a laser power of about 5 kW⋅cm^-2^ in order to minimize photobleaching as well as cell damages, stress and thus movements.

Using a home-made control software, we acquired a confocal image of the mid-section of the whole cell using CLSM, and up to 5 random positions were selected in the nucleus of the living cell, avoiding the nucleoli. We then performed autocorrelation measurements of six runs of ten seconds each for every single measurement point. A second image was recorded after the completion of the FCS measurements in order to check for cell movements. We studied 53 representative points for the EYFP construct in the nuclei of 12 different cells.

Before data analysis, a first selection was done on the basis of the recorded image: each cell showing a motion between the first and the second picture was discarded. The second selection criterion was the analysis of the recorded autocorrelation curves. For data showing inconstancy between the runs of a given point, the incriminated runs were discarded, these being often indicators for conformational changes in the cell. For measurements where more of 50 % of the runs showed inconstancy, the whole point was discarded. This procedure ensures that the recorded and analyzed fluorescence fluctuations are due to particle diffusion and not to cell movement or intracellular reorganization.

For every work session, the setup was calibrated using a 20 nM Alexa 488 solution (Molecular Probes, Eugene, Oregon) for the green channel and the focal volume was determined from the measured diffusion times.

**FFM Data analysis**

The autocorrelation curves of each individual measured point are fitted by a normal diffusion model, as described in [8]. We chose to fit the data to a normal diffusion model rather to an obstructed diffusion model because the former is more robust and allows the fit of two distinct diffusive fluorescent populations [7].

In the normal diffusion model, the mean square displacement <x2> depends linearly on the time t :

*<x^2^>* = 6 ⋅ *D* ⋅ *t* (Eq. 6)

where *D* is the diffusion coefficient.

To account for non-fluorescent processes and diffusing components of different sizes, we fitted the model function (Eq. 7) for two fluorescent diffusive components and one non-fluorescent component by the program Quickfit, written in Dr. J. Langowski laboratory and based on the Levenberg-Marquardt algorithm [9].

$G\left(\tau\right)=1+\frac{1}{N\left(1-\overline{T_{Eq}}\right)}\cdot\left[1-\overline{T_{Eq}}\cdot\left(1-e^{\frac{-\tau}{\overline{\tau_{T}}}}\right)\right]\cdot\sum_{i} \left[a{}_{i}\cdot\left(1+\frac{\tau}{\tau_{diff}}\right)^{-1}\cdot\left(1+\frac{\tau}{\tau_{diff}\cdot k_{sp}^{2}}\right)^{-\frac{1}{2}}\right]$ (Eq. 7)

In equation (7), the nonfluorescent components are due to transitions of the fluorescent molecules into the triplet state, where *T¯_Eq_* is the fraction of particles in this state, *ai* the relative fraction of each diffusive species, *N* the average number of particles in the focus volume and *k_sp_* the structure factor, which depends on the focus volume and is given by:

*k*_sp_ = *z_0_ / w_0_* (Eq. 3)

where *z_0_* is the axial and *w_0_* the lateral dimension of the detection volume. The diffusion time *τ_diff_* is related to the diffusion coefficient *D* by:

*τ_diff_ = (w_0_)2/4D* (Eq. 4)

For all measurement points we obtained the fraction of the fast and the slow components as well as their respective diffusion times. From the latter we calculated diffusion constants using the measured diffusion times of Alexa 488 at 25 °C and 37 °C and its published diffusion constant [10] as described in [7].

**Virtual Cell Modeling of the PAGFP-VP2 data**

The Virtual Cell software was used to model the PAGFP-VP2 diffusion behaviour. The nuclear geometry was similar to the one used in the FRAP simulations. The simulation time step was set to 10 ms and images were collected with 500 ms intervals. The activation of the PAGFP-VP2 was simulated by laser induced reaction where dark mobile and immobile capsids reacted with activation laser light and formed bright mobile and immobile capsids. The activation time was 3 s, as in the photoactivation experiments. Bleaching caused by the imaging was simulated by laser light induced reaction where bright mobile and immobile capsids turned back to dark mobile and immobile capsids. The total simulation time was 66 s. The reaction map is shown in supplementary Figure S5.

**Image processing and deconvolution**

The raw images from the microscopes were processed with the ImageJ software (Rasband, W.S., ImageJ, U.s National Institutes of Health, Bethesda, Maryland, USA, http://rsb.info.nih.gov/ij/). Before the measurements, the FRAP data were convolved with a 3x3 Gaussian kernel and the fluorescence was measured from the region of interest. The nuclear volume was measured by segmenting the nucleus based on the H2B-EYFP intensity. ImageJ command “Fill Holes” was used before the volume measurement to fill the space occupied by nucleoli and interchromosomal spaces. The measurement was conducted with the ImageJ plugin “3D particle analyzer”. The results were exported to Excel, to calculate the average volume of the nuclei.

Deconvolution was performed with Huygens Essential software (SVI, The Netherlands), using a theoretical PSF and the signal-to-noise ratio was approximated to 5-8. The quality threshold was set to 0.08-0.1.

For publication purposes, only linear adjustments of the image brightness and contrast were performed, avoiding clipping. The gray scale images were colored with appropriate look up table and converted to RGB images. The image finalization without brightness or contrast adjustments were performed with Photoshop CS2 (Adobe, San Jose, USA).

References

1. Phair RD, Misteli T. (2000) High mobility of proteins in the mammalian cell nucleus. Nature 404(6778): 604-609.

2. Sprague BL, Pego RL, Stavreva DA, McNally JG. (2004) Analysis of binding reactions by fluorescence recovery after photobleaching. Biophys J 86(6): 3473-3495.

3. Ihalainen TO, Niskanen EA, Jylhava J, Turpeinen T, Rinne J, et al. (2007) Dynamics and interactions of parvoviral NS1 protein in the nucleus. Cell Microbiol 9(8): 1946-1959.

4. Schaff J, Fink CC, Slepchenko B, Carson JH, Loew LM. (1997) A general computational framework for modeling cellular structure and function. Biophys J 73(3): 1135-1146.

5. Wachsmuth M. (2001) Fluoreszenzfluktuationsmikroskopie: Entwicklung eines prototyps, theorie und messung der beweglichkeit von biomolekülen im zellkern. .

6. Tewes M. (2001) Fluorescence correlation spectroscopy module for a microscope. european patent. .

7. Dross N, Spriet C, Zwerger M, Muller G, Waldeck W, et al. (2009) Mapping eGFP oligomer mobility in living cell nuclei. PLoS ONE 4(4): e5041.

8. Weidemann M.W., Tewes M., Rippe K., Langowski J. (2002) Analysis of ligand binding by two-colour fluorescence cross-correlation spectroscopy. Single Mol 3: 49-11.

9. Tewes M. (1998) Aufbau eines experimentes zur Fluoreszenzkreuzkorrelationsspektroskopi​e, erweiterung der theoretischen grundlagen und biologische anwendungen. .

10. Petrasek Z, Schwille P. (2008) Precise measurement of diffusion coefficients using scanning fluorescence correlation spectroscopy. Biophys J 94(4): 1437-1448.

**Supplementary Figure Legends:**

**Figure S1. Effect of DNaseI treatment on non-infected and infected NLFK cells**

Fixed cell confocal microscopy images of dsDNA (DAPI label), chromatin (H2B-EYFP), NS1-deYFP and PCNA-EYFP after permeabilization and treatment with buffer or DNaseI. Unapparent nuclei are encircled. Scale bars, 5 µm.

**Figure S2. Intracellular localization of capsids and viral VP proteins in PAGFP-VP2 expressing cells**

NLFK cells expressing PAGFP-VP2 were immunolabelled with antibodies that recognize intact capsids or VP proteins. Distribution of PAGFP-VP2 (green) together with (A) capsid MAb (red) or (B) VP Ab (red). Secondary antibodies used were Alexa-555-labled anti-mouse IgG and anti-rabbit IgG. Scale bars, 5 µm.

**Figure S3. Fluorescence *in situ* labelling of viral genome**

Infected cells were labelled with CPV genome specific FISH probe at 24h p.i. The FISH probe labelled the viral replication compartment inside the nucleus. Scale bar, 5 µm.

**Figure S4. Schematic presentation of NS1-deYFP Virtual Cell model**

Schematic representation of the Virtual Cell simulation showing the molecular species (green) and reactions between them (yellow). In the model, fluorescent NS1-deYFP (NS1) reacts with the viral genome (CPV_Genome) and forms genome bound NS1 (Bound NS1). Similar reaction takes place between bleached, non-fluorescent NS1 (Bleached NS1) and the viral genome. This reaction forms non-fluorescent bound NS1 (Bleached Bound NS1). Bleaching laser induces the bleaching reaction, where fluorescent NS1 forms non-fluorescent bleached NS1 or where bound NS1 forms bleached, bound NS1. Imaging laser reacts with fluorescent forms of NS1 and simulates the bleaching reaction caused by the confocal imaging.

**Figure S5. Schematic presentation of PAGFP-VP2 Virtual Cell model**

Schematic representation of the molecular species (green) and reactions between them (yellow) in PAGFP-VP2 activation study simulations. Activation laser reacts with dark, mobile and immobile capsids and leads to formation of bright capsids. Imaging laser simulates the bleaching caused by the confocal imaging.

**Figure S6. Intracellular distribution of EYFP and histone H2B in infected and non-infected cells**

Confocal images show the distribution of EYFP and H2B-EYFP in (A) in infected and (B) non-infected cells at 24 h p.i. Line profile analysis revealed intensity profiles through the nuclear region. Scale bar, 5 µm.

**Figure S7. Free EYFP and EGFP diffusion in nuclei of living cells**.

Diffusion time of free EYFP was measured in the nucleoplasm of living NLFK cells at various positions. (A) Representative NLFK cell showing 3 measurement points and (B) the measured autocorrelation curves, respectively. (C) Summary of measured EYFP diffusion coefficients in NLKF cell nuclei and EGFP diffusion coefficients in HEK293, HeLa, T98G and TP366 nuclei. Scale bar, 5 µm.

**Movie M1. Virus infection induced chromatin marginalization.**

Stably ECFP-H2B expressing cells were infected and imaged from 5 h p.i. to 24 h p.i. At 16-24 h p.i. clear increase in interchromosomal space was evident.
